# Pretreatment Assessment of Occult Uterine Sarcoma Risk Before Uterus-Preserving Treatment of Presumed Fibroids: A Narrative Review

**DOI:** 10.3390/life16101738

**Published:** 2026-10-16

**Authors:** Iustina Petra Solomon Condriuc, Raluca Anamaria Mogos, Razvan Popovici, Daniela Roxana Matasariu, Eric Oliviu Cosovanu, Catalin Mihai Buzduga, Ioana Sadiye Scripcariu, Demetra Socolov, Alexandru Carauleanu

**Affiliations:** 1 Grigore T. Popa University of Medicine and Pharmacy, 700115 Iasi, Romania; condriuc.iustina-petra@d.umfiasi.ro (I.P.S.C.); razvan.popovici@umfiasi.ro (R.P.); daniela.matasariu@umfiasi.ro (D.R.M.); catalin.buzduga@umfiasi.ro (C.M.B.); ioana.scripcariu@umfiasi.ro (I.S.S.); demetra.socolov@umfiasi.ro (D.S.); ale.carauleanu@umfiasi.ro (A.C.); 2 Department of Obstetrics and Gynecology, Cuza Voda Hospital, 700038 Iasi, Romania

**Keywords:** uterine sarcoma, uterine leiomyosarcoma, uterine leiomyoma, magnetic resonance imaging, diffusion-weighted imaging, diagnostic accuracy, uterus-preserving treatment, risk assessment, morcellation

## Abstract

Among uterus-preserving treatments for presumed fibroids, only myomectomy yields a specimen. Occult uterine sarcoma is uncommon, but leiomyosarcoma is clinically aggressive and seldom recognised before treatment. This narrative review examines what pretreatment assessment can and cannot establish. Literature from PubMed/MEDLINE, Scopus, Web of Science, Embase, and the Cochrane Library was synthesised narratively. Reported frequencies of unexpected sarcoma differ between clinical populations, mostly from 0.1% to 0.4%, and rise with age. Other predisposing factors are imprecisely quantified; genome-wide association data do not discriminate sarcoma from leiomyoma. Separate meta-analyses, largely of retrospective, enriched studies, report higher accuracy for magnetic resonance imaging (MRI), including diffusion-weighted sequences, than for ultrasound, by indirect comparison. Serum markers, composite scores and circulating nucleic acids remain supportive or investigational; directed biopsy lacks validation in unselected populations. In all-sarcoma scenarios, published likelihood ratios indicate that a negative MRI lowers, but does not eliminate, the probability of sarcoma; an exploratory, assumption-dependent reconstruction from a consecutive cohort illustrates their uncertain transportability. No available test reliably excludes sarcoma; after specimen-free therapy, a missed sarcoma may remain unrecognised until reassessment. We propose a conceptual framework, not a validated decision rule, that considers absolute residual risk alongside the specimen status of the chosen treatment.

## 1. Introduction

Uterine leiomyomas are the most common neoplasm of the female genital tract, and the range of reported occurrence is itself informative. A systematic review of registry and observational data reported incidence between 217 and 3745 cases per 100,000 woman-years and prevalence between 4.5% and 68.6%, the spread reflecting differences in study population and in whether the diagnosis was made clinically or by imaging; Black race was the only risk factor recurrently reported to increase risk, by two- to three-fold relative to White race [1]. Symptomatic disease therefore presents in very large absolute numbers, and a substantial proportion of affected women are managed without hysterectomy. Uterine sarcoma, by contrast, is rare, and its burden cannot be read from global cancer statistics. The International Agency for Research on Cancer (IARC) of the World Health Organization (WHO) estimated 434,620 new cases of cancer of the corpus uteri and 100,680 deaths worldwide in 2024, but these estimates are reported by anatomical site without subdivision by histological type, and the accompanying projection to 2050, which applies current rates to demographic forecasts, is given for all cancers combined [2]. Because the corpus uteri category consists predominantly of endometrial cancer [3], these figures cannot serve as a surrogate for the burden of uterine sarcoma or leiomyosarcoma. We did not identify a WHO or IARC estimate of the prevalence of uterine sarcoma or a sarcoma-specific projection, and extrapolating one from corpus uteri data would not be defensible. Population-based registries give the most direct estimates: in Sweden between 2010 and 2020, the crude incidence of uterine sarcoma was 1.43 per 100,000 woman-years (1.16 per 100,000 after age standardisation to the European population), carcinosarcoma being excluded because it is now considered an endometrial cancer, and leiomyosarcoma accounted for 52% of cases [4].

In this review, uterus-preserving treatment denotes any management that leaves the uterus in place: myomectomy by any route, uterine artery embolisation (UAE), radiofrequency ablation (RFA), high-intensity focused ultrasound (HIFU), and pharmacological suppression. Uterus-preserving is not the same as fertility-preserving. The term pretreatment is used rather than preoperative because embolisation, ablation and medical therapy are not operations. These treatments are not diagnostically equivalent: myomectomy delivers a specimen to the pathologist, embolisation and ablation do not, and pharmacological therapy does not provide histological resolution of the diagnostic question. The evidence on pretreatment sarcoma risk, by contrast, was assembled around a different question, whether a uterus destined for removal should be extracted intact or in fragments.

That question acquired urgency because uterine leiomyosarcoma is uncommon but clinically aggressive, and because it is rarely recognised before operation. In a regional series of 106 histologically confirmed uterine sarcomas, 65% of leiomyosarcomas had been diagnosed preoperatively as benign leiomyoma [5]. Stage at diagnosis is strongly associated with outcome: among 1396 women with uterine leiomyosarcoma in the Surveillance, Epidemiology, and End Results database, 5-year disease-specific survival was 75.8% for stage I, 60.1% for stage II, 44.9% for stage III, and 28.7% for stage IV [6]. That series was staged before a sarcoma-specific International Federation of Gynecology and Obstetrics system was introduced in 2009, so the gradient rather than the individual figures is the point. A tumour disrupted during removal may be disseminated within the peritoneum; one left in situ and treated as benign does not receive sarcoma-directed treatment until the diagnosis is eventually established.

Regulatory and professional bodies responded largely by restricting one technique. The US Food and Drug Administration (FDA) recommends that laparoscopic power morcellation be performed only within a legally marketed containment system and that it not be used in women over 50 years of age or postmenopausal women [7]; national guidance in the United Kingdom applies the same age and menopausal criterion [8]. Yet morcellation is a method of extraction, and the women who most often keep their uterus are not necessarily those in whom extraction is the operative issue. Embolisation and ablation raise the same diagnostic problem in a more absolute form, since no tissue is obtained at all.

Much of this difficulty has a biological basis. Both tumours show smooth muscle differentiation, but in most cases they follow different genetic routes: *MED12* exon 2 mutations dominate leiomyoma, whereas loss of *TP53*, *RB1*, and *ATRX* characterises leiomyosarcoma [9,10,11]. No clinically established precursor lesion has been demonstrated for uterine leiomyosarcoma, so surveillance cannot intercept a transition. Every pretreatment test must instead infer malignancy from tissue properties that the two tumours partly share: cellularity, water diffusion, necrosis, and vascularity. These properties set the limits of what imaging and circulating biomarkers can detect. They also explain why a test that performs well in selected series may perform less well in unselected women.

Several aspects of this field have been reviewed in detail: the prevalence of occult sarcoma at fibroid surgery, the imaging and laboratory features distinguishing leiomyosarcoma from leiomyoma [12], and the oncological consequences of morcellation. Decision analyses have also modelled the balance of surgical and oncological harm using published prevalence estimates [13]. The step connecting these literatures has received less attention. Pooled sensitivity and specificity are reported for magnetic resonance imaging (MRI) and ultrasound, and prevalence estimates are reported separately. The two are seldom combined to state what residual risk a negative test leaves at a given baseline risk, in the absolute terms in which consent is obtained. Nor is the analysis usually stratified by whether the planned treatment will generate a histological specimen, although that feature influences whether a missed diagnosis is likely to be recognised within days or only after years.

This review addresses that gap. It examines how the pathology of uterine smooth muscle tumours limits pretreatment discrimination, summarises the reported performance of imaging and other tests with attention to the target condition of each study, translates published likelihood ratios into residual absolute risk across representative baseline-risk scenarios, and sets that residual risk against whether the chosen treatment yields tissue for examination. The resulting framework is conceptual; it is not a validated prediction model or decision rule.

## 2. Literature Search Strategy

This narrative review drew on literature identified through PubMed/MEDLINE, Scopus, Web of Science, Embase, and the Cochrane Library. The search focused on clinically relevant evidence concerning presumed uterine leiomyoma and occult uterine sarcoma. Search terms covered uterine leiomyoma, fibroids, uterine sarcoma, and leiomyosarcoma. Additional terms addressed preoperative diagnosis, imaging, biomarkers, biopsy, morcellation, myomectomy, uterine artery embolisation, ablation, and focused ultrasound. Only English-language publications were considered. Greater weight was given to current society guidelines, regulatory documents, systematic reviews, meta-analyses, randomised trials, and large cohort studies. Relevant seminal and mechanistic studies were also identified from the reference lists of key publications.

The review focused on three main areas of evidence. These were the reported frequency of uterine sarcoma or leiomyosarcoma in women treated for presumed leiomyoma, the performance of preoperative diagnostic approaches, and outcomes after treatment of an occult uterine sarcoma. Additional targeted searches were used when needed to cover interventional radiology and device-related guidance on uterine artery embolisation, radiofrequency ablation, and focused ultrasound. Case reports were included only when they provided clinically relevant information that was not adequately addressed in larger studies.

Where reports overlapped, as with successive publications from one focused-ultrasound programme or regulatory analyses that pooled published series, the report with the most complete data for a given quantity was used and overlapping estimates were not combined. Discordant estimates are reported alongside their populations and methods, and the target condition and pathological composition of each quantitative estimate are described as reported by the source. No formal risk-of-bias assessment was undertaken, and no new pooled estimates were calculated; the calculations in Section 6 apply published summary statistics to representative baseline-risk scenarios and are presented as illustrative scenario calculations, not as a meta-analysis. SANRA was used as a framework for the transparency and quality of narrative reporting, not as a systematic-review method; the location of each element it addresses is given in Table S3 [14].

## 3. Baseline Risk of Occult Uterine Sarcoma

### 3.1. Pooled Estimates and the Reasons They Disagree

Published estimates of occult uterine sarcoma at surgery for presumed leiomyoma span more than an order of magnitude, and the variation reflects substantive differences in study populations, target definitions and study methods. A Bayesian meta-analysis of 133 studies estimated 0.51 leiomyosarcomas per 1000 procedures (95% credible interval 0.16–0.98), approximately one in 2000; restricted to the 64 prospective studies it fell to 0.12 per 1000, approximately one in 8300 [15]. The regulatory quantitative assessment published in 2017 reported a random-effects sarcoma prevalence of 0.277%, with a 95% confidence interval (CI) of 0.172% to 0.445%, and a leiomyosarcoma prevalence of 0.134% (95% CI 0.089–0.202%) [13]. Population-based data give 3.60 per 1000 for sarcoma (95% CI 2.97–4.23) and 2.33 per 1000 for leiomyosarcoma (95% CI 1.83–2.84) among 34,728 hysterectomies for leiomyomas [16], against unexpected sarcoma in 0.13% of hysterectomies for benign indication (95% CI 0.04–0.31%) but in 0.39% of operations for fibroids in a county-wide cohort [17].

Four recurring sources of heterogeneity are apparent, and each has clinical as well as methodological consequences.

Denominators differ first. Estimates derived from all hysterectomies, from hysterectomies for fibroids, from myomectomies, and from women referred for uterus-preserving treatment are not interchangeable. In a Norwegian department, 26 leiomyosarcomas were identified among 4791 women referred with anticipated benign fibroids (0.54%), but most had been suspected before surgery. A regulatory re-analysis counted six as occult (0.13%) [13,18], and unintended morcellation occurred in only one woman [18]. A Greek series makes the same point in its extreme form: all six leiomyosarcomas among 803 fibroid operations were characterised as suspicious preoperatively, and none was morcellated; the same series also contained five endometrial stromal sarcomas [19]. The prevalence of occult disease is therefore partly a property of how carefully the population was triaged.

Numerators differ as well. Some series count all uterine sarcomas, some only leiomyosarcoma, and some all uterine malignancies including endometrial carcinoma. Among 26,643 women operated on for presumed leiomyoma in China, 88 unexpected uterine sarcomas were identified (0.33%), of which 29 were leiomyosarcoma, 48 endometrial stromal sarcoma, and 11 adenosarcoma [20]. Because endometrial stromal sarcoma and adenosarcoma differ from leiomyosarcoma in presentation and in preoperative detectability, a pooled sarcoma figure overstates the leiomyosarcoma-specific risk that dominates decisions about morcellation and ablation.

Study design and era add further variation. Retrospective series depend on the completeness of pathology retrieval and on how a “presumed fibroid” was defined at the time; the difference between the full and prospective-only estimates in the meta-analysis discussed above is roughly four-fold [15]. Series conducted after the morcellation warnings tend to report intermediate values, and those assembled in referral centres tend to report higher ones, for the reasons given above.

Case mix is another important source of variation and is considered next.

### 3.2. Age, Menopausal Status, and Tumour Characteristics

Age is the most consistently reported modifier, but the available figures are of different kinds. Combining a myomectomy cohort of 2075 patients with published series, one analysis derived an aggregate uterine sarcoma risk of 2.94 per 1000 (95% CI 1.8–4.1). This figure is the sum of separate estimates for leiomyosarcoma (1.78 per 1000) and other sarcomas (1.16 per 1000), and the same analysis modelled a gradient from fewer than one in 500 in women under 30 years to 10.1 per 1000 at 75–79 years [21]. Observed age-stratified data point higher: in the five studies that stratified by age, summarised graphically by the FDA, rates of occult sarcoma among women undergoing surgery for presumed fibroids approached 2–3% after the age of 60 years; no pooled estimate was given [13]. Menopausal status points the same way, with unexpected sarcoma in 0.35% of premenopausal women (three of 851) against 0.57% of peri- and postmenopausal women (one of 174) undergoing surgery for fibroids; that contrast rests on four events and was not statistically significant [17]. Uterine weight tracked risk more steeply still, from 0.03% below 250 g to two of 13 women with a uterus of 2000 g or more, an estimate resting on 13 patients and measured on the specimen, so pretreatment use depends on an imaging estimate of uterine size [17]. None of these figures amounts to a validated individual risk calculator.

Clinical variables are informative but imprecise. One case-control study drew on 18,218 women operated on for a uterine mass, among whom sarcoma was diagnosed in 68 (0.37%). Adjusted odds ratios (ORs) were 3.30 (95% CI 1.29–8.43) for age of 40 years or more, 8.57 (95% CI 2.38–30.82) for postmenopausal status, 35.35 (95% CI 2.94–425.13) for postmenopausal bleeding, and 3.39 (95% CI 1.40–8.23) for abnormal uterine bleeding. A palpable mass carried an adjusted OR of 4.50 (95% CI 1.78–11.36), a rapidly growing mass 6.91 (95% CI 2.08–22.91) and a single sonographic mass 4.70 (95% CI 1.91–11.60) [22]. Several of these intervals constrain little: the one around postmenopausal bleeding spans a more than hundredfold range and rests on very few events, so its point estimate should not be read as a stable multiplier. These odds ratios describe case-control associations; they cannot be converted into individual absolute risks without a validated prediction model.

Rapid growth deserves separate mention because the evidence is divided. Of 1332 women operated on for presumed leiomyoma, 371 had rapid uterine growth; one leiomyosarcoma occurred in that subgroup, and none of the 198 meeting a published definition of rapid growth had a sarcoma; overall sarcoma incidence was 0.23% [23]. That study predates modern imaging and had limited power to exclude a small effect. In the case-control analysis above, by contrast, a rapidly growing mass carried an adjusted OR of 6.91 [22]. The two findings are compatible if growth matters mainly where it is unexpected, and UK consent advice identifies rapid growth of a solitary presumed fibroid in perimenopausal women aged 50 years or younger as a feature associated with greater concern for sarcoma [24]. In premenopausal women, growth remains a weak discriminator.

One denominator deserves separate mention because it describes a population in which uterus-preserving treatment is actually delivered. Among 15,759 consecutive women presenting for high-intensity focused ultrasound of presumed fibroids, all of whom underwent pretreatment MRI, 17 uterine sarcomas were ultimately identified (0.108%); 12 were low-grade endometrial stromal sarcomas and five leiomyosarcomas [25]. An extension of the same programme to 41,631 women in 13 hospitals identified 27 occult sarcomas [26].

The estimates in Table 1 are best read as representative baseline-risk scenarios from different clinical populations rather than as one universal prevalence range. For uterine sarcoma as a whole, contemporary series in populations comparable to those considered for uterus-preserving treatment cluster between about 0.11% and 0.39%, with modelled and observed values above 1% in the oldest surgical populations. Estimates reported specifically for leiomyosarcoma run lower, from 0.012% in pooled prospective data to 0.233% in population-based data. Higher figures of 0.54% and 0.75% come from referral series in which most cases were suspected preoperatively rather than occult. Most underlying cohorts underwent hysterectomy and are older than the women who present for uterus-preserving treatment, so they may overestimate the risk in younger women selected for such treatment. Most also count sarcoma that was unsuspected after the preoperative assessment in use at the time, so they are partly post-test rather than pre-test quantities, a point that is central to the interpretation of Section 6. Because the diagnostic syntheses in Section 5 are framed around uterine sarcoma, the all-sarcoma scenarios are carried forward.

### 3.3. Predisposing Factors Beyond Age and Menopausal Status

The clinical features considered in Section 3.2 (abnormal bleeding, uterine size, growth and a solitary mass) describe a tumour that is already present and act as discriminators at presentation. Predisposing factors are of a different kind: they alter the probability that a sarcoma has developed, and none has been incorporated into a validated pretreatment model. UK consent advice lists a history of tamoxifen use, a history of pelvic irradiation, ethnicity, and a family history of breast or ovarian carcinoma, a *BRCA1/2* germline variant or Lynch syndrome among its red-flag indicators [24]. The evidence behind these factors is limited. The association with tamoxifen is best quantified for malignant mixed Müllerian tumour, observed 4.62 times as often as expected among women treated with tamoxifen for breast cancer [27]; that tumour is carcinosarcoma, now considered an endometrial cancer [4], and we did not identify an estimate specific to leiomyosarcoma. Pelvic irradiation had preceded 13 of 114 uterine sarcomas, by a median of 17 years, in an institutional series from 1955 to 1987 [28]. Both exposures are uncommon among women presenting with presumed fibroids.

Race requires careful interpretation. In US registry data for women older than 35 years, the incidence of uterine leiomyosarcoma was 1.51 per 100,000 in Black women and 0.91 per 100,000 in White women [29]. Black women also carry a two- to three-fold higher risk of fibroids [1], and in the integrated health system cohort of hysterectomies for leiomyomas, occult sarcoma was not more frequent in African-American women (Section 9.3) [13,16]. A higher population incidence therefore does not establish a higher risk for an individual presumed to have a fibroid. Obesity and diabetes were associated with uterine sarcoma other than carcinosarcoma in a pooled analysis of 229 cases, with odds ratios of 1.73 and 2.33 [30]. Both are common and are also risk factors for endometrial carcinoma, and the subtype analyses, based on 98 endometrial stromal sarcomas and 82 leiomyosarcomas, were imprecise [30].

Hereditary predisposition is uncommon but specific. Uterine leiomyosarcoma developed in seven of 525 women with hereditary retinoblastoma in one cohort, a substantial excess over population rates [31], and germline *TP53* variants (Li–Fraumeni syndrome) predispose to leiomyosarcoma, although predominantly at soft-tissue sites [32]; these and other genetic data are considered in Section 5.6. A relevant exposure or syndrome warrants the careful discussion that UK consent advice asks for [24], but it does not discriminate between sarcoma and leiomyoma in an individual woman.

## 4. Why Pretreatment Discrimination Is Intrinsically Difficult

### 4.1. Three Diagnostic Categories with Overlapping Appearances

The fifth edition of the World Health Organization classification of female genital tumours integrates morphology with molecular events, and many of the major changes relevant to gynaecological mesenchymal tumours concerned uterine and vulval tumours [33]. For the diagnostic problem considered here, uterine smooth muscle tumours can be organised into three clinically relevant groups: leiomyoma and its variants, smooth muscle tumour of uncertain malignant potential (STUMP), and leiomyosarcoma. Leiomyoma subtypes, including cellular, mitotically active, epithelioid, myxoid and fumarate hydratase (FH)-deficient leiomyoma, and leiomyoma with bizarre nuclei, are benign. Several nevertheless reproduce, on imaging and occasionally on the resected specimen, the features by which malignancy is recognised. 

STUMP is instructive because it is defined by what cannot be established. It is a diagnosis of exclusion, reached only after adequate sampling and expert review against the established parameters of cytological atypia, mitotic index and tumour cell necrosis, and applied when a tumour meets neither the criteria for leiomyoma nor those for leiomyosarcoma [34]. In a series of 16 STUMPs followed for 21 to 192 months, two recurred, at 15 and 51 months [34]. A later series of 22 cases reported adverse outcomes in eight (36.4%), against 7–26.7% in previously published reports, a spread its authors attributed to the different criteria applied [35]; referral enrichment is at least as plausible an explanation, since consultation series are assembled from the cases that proved difficult. Variation in historical diagnostic criteria, sampling, and referral enrichment contribute to the wide range of reported STUMP outcomes, limiting the usefulness of STUMP as a stable target for pretreatment prediction.

Intraoperative frozen section has been proposed as a partial safeguard. In 112 frozen sections of uterine smooth muscle tumours, comprising nine leiomyosarcomas and 103 leiomyomas, frozen and permanent sections agreed in 99 cases (88%), with false positives in 0.9% and no false negatives; diagnosis was deferred in 10 cases (8.9%), and the principal errors involved misinterpretation of stromal hyalinisation as tumour cell necrosis [36]. Those figures are reassuring, but the series was heavily selected: frozen section was requested because of an abnormal gross appearance or intraoperative discovery of abnormal growth in more than half of cases. Its performance in an unselected fibroid population, where the gross appearance is by definition unremarkable, is unknown.

### 4.2. Molecular Evidence Against a Progression Sequence

The genomic evidence does not describe a simple progression. Exome and targeted sequencing identified alterations of *MED12* exon 2 in 70% of uterine leiomyomas [9]. Prospective clinical sequencing of 107 uterine sarcomas found a different mutational profile in the 80 leiomyosarcomas: loss-of-function alterations of *TP53* in 56%, *RB1* in 51% and *ATRX* in 31%, and homozygous *BRCA2* deletion in 5%; whole-genome duplication was present in 50.5% of the high-grade tumours in the series as a whole [10]. The two sets of drivers overlap little, which is consistent with largely separate origins rather than malignant transformation of an existing leiomyoma. That inference should not be pressed far. It rests on cross-sectional comparison, and direct counter-evidence exists: sequencing of 41 uterine leiomyosarcomas found somatic *MED12* exon 2 mutations in three of them, 7%, leading those authors to propose that a subgroup may develop from a leiomyoma precursor [11]. Three events cannot establish a pathway, but they show the two populations are not disjoint.

In epithelial malignancies of the female genital tract, screening exploits a detectable precursor lesion; no such precursor has been established for uterine leiomyosarcoma. STUMP is not established as a precursor lesion; it is a category of diagnostic uncertainty defined on resected tissue rather than a lesion identified and removed before malignancy. Without a precursor, the logic of watchful waiting weakens: an interval scan can detect growth, but growth is an inconsistent marker of malignancy [22,23].

Fumarate hydratase-deficient leiomyoma illustrates misclassification in the opposite direction. It is a recognised benign subtype in the current classification [33], yet its appearance can be alarming enough to raise the question of malignancy. Recognising it matters beyond the uterus, since the morphology may be the first indication of fumarate hydratase (FH) tumour predisposition syndrome, historically termed hereditary leiomyomatosis and renal cell cancer, a syndrome whose consequences fall outside gynaecological care.

### 4.3. Shared Tissue Properties and Imaging Overlap

**Figure 1:**
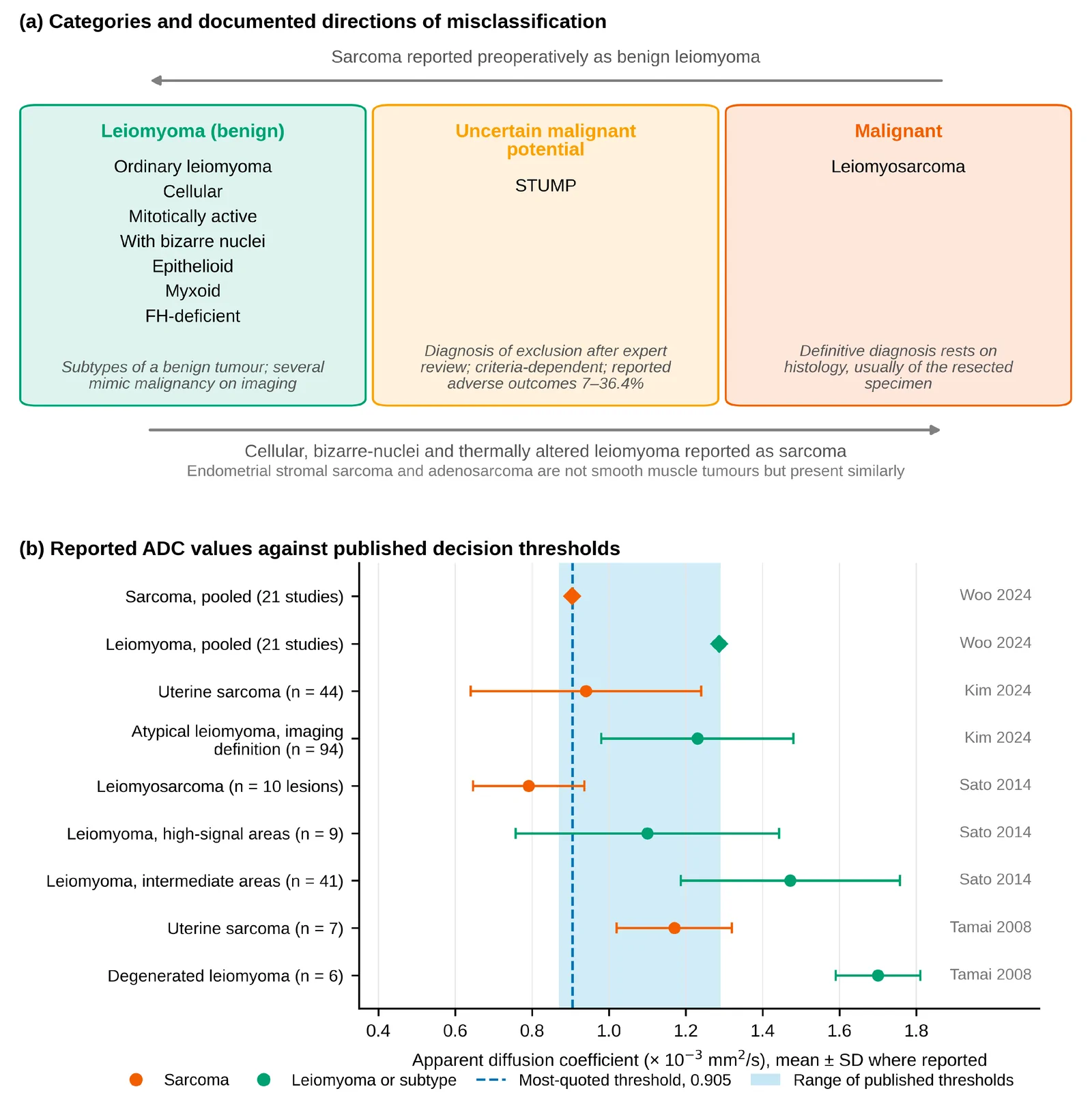
Sources of pretreatment diagnostic uncertainty in uterine smooth muscle tumours. (**a**) Three clinically relevant diagnostic groups of uterine smooth muscle tumours, based on the current World Health Organization classification; selected leiomyoma subtypes, and the two directions in which misclassification has been documented: sarcoma reported preoperatively as benign leiomyoma, and cellular, bizarre-nuclei, or thermally altered leiomyoma reported as sarcoma [5,33,35,39]. Endometrial stromal sarcoma and adenosarcoma are not smooth muscle tumours but present in the same clinical way and are therefore relevant to the same decisions. (**b**) Mean apparent diffusion coefficient by tumour class as reported in the source studies, shown as mean ± standard deviation where reported and as a point estimate otherwise, against the most widely quoted decision threshold and the range of thresholds reported across 21 studies [37,40,41,42,43]. In one study, sarcomas also overlapped with ordinary and cellular leiomyomas [37]. Vermilion denotes sarcoma and green denotes leiomyoma or a recognised leiomyoma subtype. Values are those published in the cited sources and have not been recalculated. ADC, apparent diffusion coefficient; FH, fumarate hydratase; STUMP, smooth muscle tumour of uncertain malignant potential.

The features that make leiomyosarcoma conspicuous on imaging are high cellularity, restricted water diffusion, necrosis and haemorrhage. None is specific. Cellular leiomyoma is hypercellular, and on diffusion-weighted imaging (DWI) it behaves accordingly: in the earliest systematic comparison, both sarcomas and cellular leiomyomas showed high signal intensity, and the mean apparent diffusion coefficient (ADC) of sarcomas, 1.17 ± 0.15 × 10^−3^ mm^2^/s, separated cleanly from normal myometrium and degenerated leiomyoma but overlapped with ordinary and cellular leiomyoma [37]. Degenerating leiomyoma contributes to necrosis and haemorrhage without malignancy; in a retrospective series of histologically proven cases, concomitant necrosis and haemorrhage occurred only in leiomyosarcoma, most leiomyosarcomas had smooth or lobulated margins like their benign counterparts, and quantitative multiparametric MRI measures did not differ significantly between groups [38]. One terminological caution belongs here: in pathology the entity formerly called atypical leiomyoma is now leiomyoma with bizarre nuclei, whereas in the radiology literature quoted below the term denotes a leiomyoma with atypical imaging features. Figure 1 summarises the diagnostic categories and documented directions of misclassification (panel a) and illustrates the overlap between reported ADC values and published thresholds (panel b), which cannot be resolved by a single cut-off.

### 4.4. From Tissue Biology to Imaging Signal

Each imaging criterion is a proxy for a tissue property. Restricted diffusion reflects cellular density, so low apparent diffusion coefficient values accompany both leiomyosarcoma and cellular leiomyoma [37,42]. Tumour cell necrosis is a defining histological criterion of malignancy, whereas ischaemic-type necrosis and hyaline degeneration occur in benign tumours and also produce non-enhancing areas; even subspecialist pathologists agree only moderately on the presence of tumour cell necrosis (κ = 0.44) [44]. High T1 signal from haemorrhage occurs in degenerating leiomyomas as well as in sarcomas [45]. Histotype adds a further layer. Myxoid leiomyosarcoma, rich in extracellular matrix, has intrinsically high apparent diffusion coefficient values and can therefore fail ADC-based consensus MRI criteria designed around conventional tumours [46]. Imaging therefore detects biological states rather than diagnoses, and its misclassification rate depends on how often benign tumours adopt those states in the population examined.

## 5. Diagnostic Performance of Pretreatment Tests

Most syntheses are framed around uterine sarcoma as a whole, although their pathological definitions differ, whereas several primary studies target leiomyosarcoma alone; these targets are not interchangeable, and applying an all-sarcoma estimate to leiomyosarcoma is an approximation, because performance may differ between histological subtypes and a pooled estimate reflects the case mix of its source cohorts. Most primary studies are retrospective, the prospective exceptions being identified in Table S2. Most compare a sarcoma group with a separately assembled leiomyoma group rather than following a consecutive cohort; in the most recent MRI synthesis, eligible patients had masses already suspected of sarcoma, and benign comparators were often atypical, T2-hyperintense or large leiomyomas [45]. The prevalence within these studies, which runs from under 2% to almost 50%, also exceeds that of the population to which the results would be applied. Spectrum and selection effects can alter apparent diagnostic performance in either direction: conspicuous sarcomas may increase apparent sensitivity, whereas atypical benign comparators may reduce apparent specificity. Predictive values are also not directly transportable to populations with different prevalence and case mix.

### 5.1. Ultrasound

In a meta-analysis of three studies including 972 women (694 with leiomyoma and 278 with sarcoma), ultrasound achieved a pooled sensitivity of 0.76 (95% CI 0.70–0.81) and specificity of 0.89 (95% CI 0.87–0.92), with a pooled positive likelihood ratio of 6.65 (95% CI 4.45–9.93) and negative likelihood ratio of 0.26 (95% CI 0.07–1.00) [47]. Its authors describe the analysis as exploratory. Heterogeneity was high for sensitivity (I^2^ 94.5%) and for the negative likelihood ratio (I^2^ 96.9%), and the upper confidence limit of the latter reached 1.00, the value at which a negative result carries no information [47].

Descriptive work using standardised terminology explains why. Comparing 16 uterine sarcomas with 91 leiomyomas, women with sarcoma were older (median 65 versus 48 years) and more often postmenopausal (13 of 16 versus 15 of 91); irregular borders, non-uniform echogenicity and abundant intralesional vascularity were common in sarcoma without being exclusive to it, and interobserver agreement was only fair to moderate [48]. A structured sonographic score developed prospectively in 545 women reached sensitivity 93.8%, specificity 97.9%, and negative predictive value 99.8%, although its positive predictive value was 57.7% even at that referral cohort’s prevalence, and it was evaluated in the cohort in which it was derived [49].

### 5.2. Magnetic Resonance Imaging

Separate evidence syntheses report higher pooled diagnostic-performance estimates for MRI than for ultrasound; the underlying study sets and populations differ, and the estimates should not be read as a direct comparison. A meta-analysis of eight studies including 2495 women reported pooled sensitivity 0.90 (95% CI 0.84–0.94) and specificity 0.96 (95% CI 0.96–0.97), with a positive likelihood ratio of 13.55 (95% CI 6.20–29.61) and a negative likelihood ratio of 0.08 (95% CI 0.02–0.32) [50]. All but one of its studies were retrospective; one surgical cohort of 1960 patients supplied most of the women, and indeterminate MRI reports were counted as positive [50,51]. A synthesis of 12 retrospective studies published between July 2019 and April 2025 reported sensitivity 84.2% (95% CI 76.3–89.9%) and specificity 89.1% (95% CI 78.1–94.9%), with I^2^ of 66.3% and 95.8% [45]. It was restricted to masses suspected of sarcoma, verified benign diagnoses by histology or by imaging or clinical follow-up, and did not report likelihood ratios [45]. Both syntheses were framed as evaluations of MRI for uterine sarcoma, but their pathological target conditions were not identical [45,50]. In the more recent synthesis, the malignant cohorts were heterogeneous and included, in addition to sarcomas, smooth muscle tumours of uncertain malignant potential, carcinosarcoma, and adenosarcoma in some contributing studies; its pooled estimates should therefore not be interpreted as strictly sarcoma-only operating characteristics [45]. The difference between them cannot be resolved from published data, and neither describes performance in unselected women.

Diffusion-weighted imaging carries much of the discriminating power. Across 21 studies, ADC measurement achieved pooled sensitivity 89% (95% CI 82–94%) and specificity 86% (95% CI 78–92%), specificity being materially higher when ADC was interpreted alongside other multiparametric features (95%, 95% CI 92–99%) than when read alone (82%, 95% CI 75–89%) [42]. Across those studies, thresholds ranged from 0.87 to 1.29 × 10^−3^ mm^2^/s without significantly different performance (*p* = 0.37), and the pooled means were 0.904 × 10^−3^ mm^2^/s for sarcoma and 1.287 × 10^−3^ mm^2^/s for leiomyoma [42]. Both pooled means lie inside the range of published thresholds, which is the quantitative statement of the overlap shown in Figure 1b.

Structured algorithms improve consistency more than discrimination. A multidisciplinary consensus statement has set out a standardised MRI approach to risk assessment of uterine masses [52]. An algorithm combining nodal or peritoneal disease, DWI signal exceeding that of endometrium, and ADC of 0.905 × 10^−3^ mm^2^/s or less achieved sensitivity 98% (95% CI 90–100%) and specificity 94% (95% CI 88–98%) in training, and 83% and 97% with a less experienced reader [43]. Independent validation in 40 examinations read by two blinded radiologists gave sensitivity 75% and 81.2% and specificity 95.8% and 91.7% for malignancy overall, with κ = 0.89 [53]. That validation rests on 16 malignancies in a single centre, so the apparent perfection of its leiomyosarcoma subgroup is compatible with substantially poorer performance elsewhere. Series closer to a screening population are few: one compared a small number of sarcomas with 1942 leiomyomas [51], and another assessed 43 women planned for focused ultrasound, 11 of whom had leiomyosarcoma or STUMP [54].

Two further observations qualify the imaging literature. A four-institution study of 138 patients showed that adding quantitative DWI to qualitative features raised the validation area under the curve from 0.78 to 0.92 (*p* < 0.001), which locates the increment in diffusion rather than morphology [41]. The ADC meta-analysis proposed a test-positive threshold of 0.904 × 10^−3^ mm^2^/s, derived from the pooled mean ADC of sarcomas [42], almost identical to the most widely quoted algorithmic threshold of 0.905 × 10^−3^ mm^2^/s [43]. Within a single population, a threshold at the centre of the sarcoma distribution would classify about half of sarcomas as benign. The coincidence is less paradoxical than it appears: the algorithm applies its threshold only after a diffusion-signal step and was derived in one population, whereas the pooled mean averages study-level means obtained with different b-values and regions of interest. Neither number can be transferred to a new setting without local calibration.

### 5.3. Serum Markers and Composite Scores

The most frequently cited biomarker result is also the least generalisable. In a prospective study of 227 eligible patients, among whom 10 had leiomyosarcoma and 130 had a degenerated leiomyoma, serum lactate dehydrogenase (LDH) with its isozymes combined with gadolinium-enhanced dynamic MRI produced sensitivity, specificity and both predictive values of 100% [55]. A point estimate of 100% based on only 10 malignant cases is highly imprecise and does not establish perfect diagnostic performance, and later multicentre work has not reproduced it [56]. A meta-analysis of 20 observational studies covering 13 biomarkers found LDH sensitivity ranging from 0.47 to 1.00 and specificity from 0.86 to 1.00, cancer antigen 125 (CA-125) sensitivity 0.43–0.64 with specificity 0.50–0.77, and C-reactive protein sensitivity 0.84–0.87 with specificity 0.83–0.89 [57]. These are ranges across studies rather than pooled estimates, and their width makes them unusable as decision thresholds.

Contemporary multicentre data are more sober. Across 15 Japanese centres, 139 sarcomas, a group that included 16 STUMPs, and 141 fibroids were compared: the routine preoperative report achieved sensitivity 77.7% with specificity 92.9%, while two prespecified MRI-and-LDH algorithms traded these against each other, at 92.1%/72.3% and 82.0%/85.8% [56]. No serum marker performed well enough to decide management alone.

Composite scores are the most promising non-imaging approach, provided their development history is read carefully. The PREoperative Sarcoma Score, or PRESS, was derived in 63 patients including 15 sarcomas and reported 84.1% accuracy with 93.2% negative predictive value [58]; its revision, reported a year later with 93.7% accuracy, was refitted on the same patients [59]. The strongest external evidence is a case-control validation using 90 new leiomyosarcomas and 659 prospectively recruited leiomyoma cases, in which a composite score designated pLMS achieved an area under the curve of 0.97 [60]. Case-control assembly may overestimate apparent discrimination because of spectrum and design effects and limits transportability to unselected clinical populations; no composite score has been tested prospectively in consecutive women presenting for uterus-preserving treatment.

### 5.4. Circulating Nucleic Acids

Circulating nucleic acids sample tumour-derived material directly rather than inferring it from tissue properties, but they are at an earlier stage and carry a specific warning. Tumour-derived copy-number alterations were detectable in the plasma of six of 12 women with ordinary leiomyoma [61]. Leiomyomas are clonal neoplasms, so tumour-derived DNA in plasma does not by itself indicate malignancy, and a naive circulating-tumour-DNA test would generate positive results in benign disease. A cell-free RNA classifier based on a 100-gene signature, developed in 102 prospectively recruited women with 78 leiomyomas and 24 leiomyosarcomas, achieved an area under the curve of 0.868 with sensitivity 0.732 and specificity 0.813 in cross-validation [62]. A sensitivity of 0.732 would not, on its own, warrant a change in management. One recent study paired a clinical model with exploratory transcriptomic analysis. In 23 postmenopausal women with uterine mesenchymal tumours and 92 matched fibroid controls, pelvic pressure, abnormal uterine bleeding, and tumour diameter gave an apparent area under the curve of 0.859; a public dataset was then used to characterise expression differences between leiomyosarcoma and fibroid tissue [63]. None of these approaches has been evaluated in consecutive women presenting for uterus-preserving treatment.

### 5.5. Tissue Sampling

Endometrial sampling is the most widely available tissue test and among the least suited to this particular task. Among 938 patients with uterine malignancy, sampling was reviewable in 730; of 142 uterine sarcomas, 72 were sampled, and sampling indicated an invasive tumour in 86% (62 of 72) but identified the correct histological type in only 64% (46 of 72), against 81% for epithelial tumours (*p* < 0.001) [64]. Both figures are conditional on a sample having been obtained; across all 142 sarcomas, the correct type was named in 46. Because endometrial stromal sarcoma and adenosarcoma involve the cavity more often than leiomyosarcoma, a leiomyosarcoma-specific sensitivity cannot be derived from these data and is likely lower. This limited yield for sarcoma, and in particular for a tumour arising in the myometrium, does not diminish the role of endometrial sampling where it is otherwise indicated, for example in women with abnormal or postmenopausal bleeding, in whom it serves to detect or exclude endometrial carcinoma and other pathology of the uterine cavity (Section 8).

Directed biopsy of the mass itself is a different proposition. Transcervical needle biopsy was performed in 435 patients with myoma-like tumours; the diagnostic-accuracy analysis included 372 biopsies from 351 patients, with seven sarcoma-positive and 365 sarcoma-negative observations. At the selected cut-off, sensitivity was 100% (95% CI 59–100%), specificity 98.6% (95% CI 97.4–99.8%), positive predictive value 58% (95% CI 30–86%), and negative predictive value 100.0% (95% CI 99.0–100.0%) [65]. Patients without surgical verification were classified using at least two years of clinical follow-up. Only seven sarcomas were present, and some patients contributed repeat biopsies, so the estimates are highly imprecise, and the 100% point estimates do not establish perfect diagnostic performance. A prospective pilot combining percutaneous needle biopsy with array comparative genomic hybridisation in 34 patients, 11 with sarcoma, reported 94% accuracy for microscopy alone and 100% combined, with no complications and no dissemination along the biopsy track [66]. Point estimates of 100% based on only seven and 11 sarcomas are highly imprecise and do not establish perfect diagnostic performance; sampling error in a heterogeneous tumour is unquantified, and none of this evidence comes from women with unremarkable imaging. These reservations concern the amount of evidence and the selection of the patients studied, not the direction of the findings, which were favourable in both series. The evidence is therefore insufficient to establish the diagnostic accuracy and clinical utility of directed biopsy in unselected populations; this should not be interpreted as evidence that the technique is ineffective. Table 2 summarises the pooled and validation-level performance estimates for the diagnostic approaches discussed above, with the target condition of each estimate specified as reported by its source.

### 5.6. Genetic and Genomic Data

Genetic data could contribute to pretreatment assessment by identifying women whose inherited predisposition raises the baseline risk or by detecting features of the tumour itself. Germline, genome-wide association, somatic, and circulating data answer different questions, and none has been evaluated in consecutive women presenting for uterus-preserving treatment. Germline testing identifies a small group at higher risk rather than characterising a given mass. In a tertiary series in which 165 women with uterine leiomyosarcoma underwent paired tumour–normal sequencing, pathogenic germline variants were found in 35 (21%), but only nine (5%) showed biallelic inactivation in the tumour, the pattern expected when the variant drives tumour development; most of these nine lay in DNA damage-repair genes, and Li–Fraumeni syndrome and hereditary retinoblastoma accounted mainly for soft-tissue rather than uterine leiomyosarcoma [32]. The authors considered these proportions slightly inflated by referral and selection [32]. Germline FH variants, which underlie FH tumour predisposition syndrome (Section 4.2), were found in one of 67 women with early-onset uterine leiomyosarcoma in a population-based series [67]. A known syndrome therefore raises the prior probability for the few women who carry one, but germline testing would not be expected to discriminate sarcoma from leiomyoma in unselected women with presumed fibroids.

Genome-wide association studies, which test common variants, have been informative for leiomyoma but not for sarcoma. The largest multi-ancestry meta-analysis of uterine fibroids included 74,294 cases and 465,810 controls [68], and an earlier meta-analysis of 16,595 cases identified risk variants in or near genes including *TP53*, *TERT*, *ATM*, and *ESR1* [69]. These loci describe susceptibility to leiomyoma and were not designed to distinguish leiomyosarcoma from leiomyoma; the earlier study reported that it lacked the power to examine leiomyosarcoma, with 44 cases available [69]. A polygenic risk score for fibroids separated women with and without fibroids only modestly, with an area under the curve of 0.56, or 0.60 after age, body mass index and ancestry were added [70]. We did not identify a genome-wide association study of uterine leiomyosarcoma or uterine sarcoma, or a polygenic score evaluated for discriminating leiomyosarcoma from leiomyoma. The rarity of uterine sarcoma and the heterogeneity of its histological types make adequately powered discovery and independent validation difficult, and susceptibility loci for benign fibroids cannot be extrapolated into a test for sarcoma.

Somatic genomics marks the clearest biological difference between the two tumours, *MED12* mutation in most leiomyomas against loss of *TP53*, *RB1*, and *ATRX* in leiomyosarcoma (Section 4.2) [9,10], but it requires tumour material. Before treatment, tissue from a myometrial mass can be obtained only by directed biopsy, with which array comparative genomic hybridisation has been combined in a small pilot study (Section 5.5) [66]; otherwise, tumour-derived material can be sampled only indirectly, in plasma (Section 5.4). Detection of circulating tumour DNA by low-coverage whole-genome sequencing depends on tumour burden: in one leiomyosarcoma series it was found in 11 of 16 patients with progressive disease and a total tumour burden above 5 cm, and in none of 16 with stable or low-burden disease [71]. Conversely, tumour-derived copy-number alterations were detectable in the plasma of six of 12 women with ordinary leiomyoma [61]. Both findings limit what plasma DNA can be expected to achieve for a localised presumed fibroid, and the cell-free RNA classifier and transcriptomic analyses described in Section 5.4 remain at an early stage [62,63].

Genetic and genomic data therefore clarify the biological differences between leiomyoma and leiomyosarcoma and may support the development of biomarkers. At present, however, no germline test, genome-wide association signal or polygenic score provides validated pretreatment discrimination of leiomyosarcoma in women with presumed fibroids; somatic and circulating assays remain investigational, and none of these data is incorporated in the framework proposed here.

## 6. From Test Performance to Absolute Residual Risk

Predictive values cannot be carried between populations, because they depend on prevalence. Sensitivity and specificity are often treated as relatively transportable between populations, and the calculation below necessarily relies on that assumption. That assumption is uncertain in this setting, because the underlying studies frequently compare selected sarcoma groups with separately assembled leiomyoma comparators, so the spectrum of disease on both sides may be unrepresentative. The derivation that follows should therefore be read as an arithmetic exercise on inputs of uncertain transportability, and it is set beside an exploratory, assumption-dependent reconstruction from a consecutive cohort below.

A second assumption concerns the pre-test probability, and it is the central limitation of this section. Most estimates in Table 1 count sarcomas that were unsuspected after the preoperative assessment in use at the time, which often included ultrasound and sometimes MRI. They are therefore partly post-test quantities and not true pre-imaging probabilities. Applying a further negative result to them assumes that the second test is conditionally independent of the first, which is unlikely for two imaging tests of the same tissue properties, and may bias the calculated residual risk downward. The focused-ultrasound cohort is closer to a true pre-test population, because its denominator includes the sarcomas that MRI subsequently flagged [25]. The baseline risks entered below are representative population-level scenarios for all uterine sarcoma, each with substantial uncertainty of its own, and not validated individual pre-imaging probabilities. The arithmetic of Bayes’ theorem is not in question; what limits interpretation is the transportability of its inputs. The outputs therefore indicate the direction and approximate size of the shift that a test result would produce in a given scenario, and they should not be read as the post-test probability of an individual woman.

**Figure 2:**
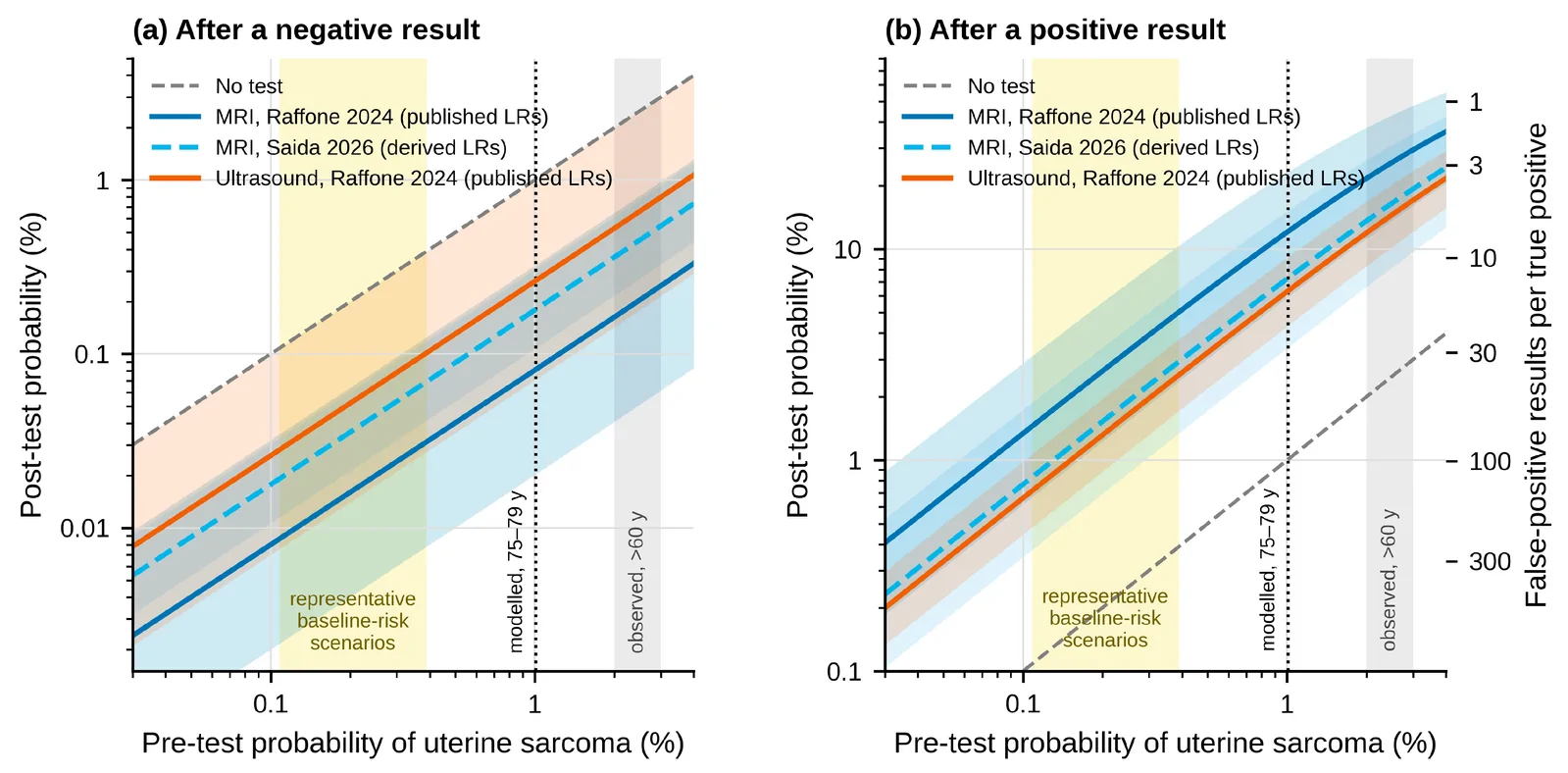
Post-test probability of uterine sarcoma (all histological types) as a function of pre-test probability: an illustrative scenario calculation, not an individualised risk calculator. (**a**) After a negative result. (**b**) After a positive result; the right-hand axis gives the number of false-positive results per true positive, (1 − *p*)/*p*, where *p* is the post-test probability. Curves apply Bayes’ theorem in odds form (post-test odds = pre-test odds × likelihood ratio). Solid lines use published pooled likelihood ratios: MRI, LR+ 13.55 and LR− 0.08 [50]; ultrasound, LR+ 6.65 and LR− 0.26 [47]. The dashed line is a secondary scenario using likelihood ratios derived from the pooled sensitivity (0.842) and specificity (0.891) of a synthesis that did not report likelihood ratios and whose malignant cohorts were pathologically heterogeneous (LR+ 7.72, LR− 0.177) [45]; this scenario is illustrative rather than strictly target-matched. Shaded areas are scenario envelopes, not formal 95% confidence or credible intervals: for published likelihood ratios they span the reported 95% confidence limits of each ratio, and for the derived scenario the extreme combinations of the marginal 95% limits of sensitivity and specificity. The comparison between MRI and ultrasound is indirect. The dashed grey line (no test) is the line of identity. The yellow band marks representative baseline-risk scenarios for all uterine sarcoma from Table 1 (0.108–0.39%); the dotted line marks the value modelled for women aged 75–79 years [21]; the grey band marks the rates of 2–3% approached after the age of 60 years in five age-stratified surgical series [13]. Both axes are logarithmic. Values are listed in Table S1. The marked baseline risks are population-level scenarios for all uterine sarcoma. Most are partly post-assessment quantities and not true pre-imaging probabilities, so the curves in panel (**a**) may underestimate residual risk (Section 6), and none of the curves gives leiomyosarcoma-specific probabilities. The figure is not a validated prediction model and defines no clinical decision threshold. LR+, positive likelihood ratio; LR−, negative likelihood ratio; MRI, magnetic resonance imaging.

The calculation uses likelihood ratios. Where a synthesis reported pooled likelihood ratios, those published values are used. For MRI, they are 13.55 (95% CI 6.20–29.61) and 0.08 (95% CI 0.02–0.32) [50]; for ultrasound, 6.65 (95% CI 4.45–9.93) and 0.26 (95% CI 0.07–1.00) [47]. Both syntheses pooled each accuracy measure separately with univariate random-effects models [47,50], so the published likelihood ratios need not equal those implied by the pooled sensitivity and specificity (22.5 and 0.10 for MRI). Such differences can arise when the accuracy measures are pooled separately [72]. The more recent MRI synthesis reported only sensitivity and specificity [45]; likelihood ratios derived from them (7.72 and 0.177) are used as a secondary scenario and are not formal pooled estimates. The published likelihood-ratio scenarios use the target conditions defined in their respective source syntheses [47,50]. The secondary MRI scenario derived from the pooled sensitivity and specificity reported by Saida et al. [45] reflects a heterogeneous malignant target that included STUMP, carcinosarcoma, and adenosarcoma in some contributing studies; applying it to baseline risks for uterine sarcoma is therefore illustrative rather than strictly target-matched. Applied to leiomyosarcoma alone, any of these estimates is an approximation, because neither the baseline risks nor the likelihood ratios are specific to leiomyosarcoma; the outputs are all-sarcoma scenarios and should not be read as leiomyosarcoma-specific probabilities. Figure 2 shows the resulting post-test probabilities, and Table S1 lists every input and output.

On the published pooled negative likelihood ratio, a negative MRI lowers the odds of sarcoma about 12.5-fold (scenario envelope 3.1- to 50-fold); on the derived secondary scenario, about 5.6-fold (3.3- to 9.4-fold). At the baseline risk observed for all uterine sarcoma in the consecutive focused-ultrasound cohort, 0.108% [25], a negative study would leave approximately 0.009% (about 1 in 11,600) or 0.019% (about 1 in 5200). At the population-based all-sarcoma figure of 0.360% for hysterectomy for leiomyomas [16], the corresponding values are approximately 0.029% and 0.064%. A negative ultrasound lowers the odds by about 3.8-fold on its published likelihood ratio, but its envelope extends to no change at all, because the upper confidence limit of the pooled negative likelihood ratio is 1.00 [47].

The consecutive focused-ultrasound cohort allows an exploratory real-world reconstruction, which is neither an external validation nor a diagnostic-accuracy study [25]. Among 15,759 women with pretreatment MRI, 46 were reported as suspicious on T2-weighted features and 11 of these proved to be sarcoma at laparotomy. Among the 15,713 who proceeded to ablation, eight developed recurrence during follow-up and six were confirmed histologically as sarcoma [25]. A two-by-two table can be reconstructed only by assuming that these six sarcomas were already present at the baseline MRI, that the 35 suspicious women without reported sarcoma were false positives, and that every other woman was truly negative. On those unverified assumptions, the reconstructed proportions corresponding to sensitivity, specificity and positive predictive value would be 11 of 17 (64.7%), 15,707 of 15,742 (99.8%), and 11 of 46 (23.9%). These are assumption-dependent descriptive quantities for all uterine sarcoma, not diagnostic-accuracy estimates, and they are not directly comparable with sensitivity and specificity obtained in formally designed diagnostic-accuracy studies. On the same assumptions, the risk after a negative study would be six of 15,713, or 0.038% (about 1 in 2600), a reduction of about 2.8-fold. Expressed per 100,000 similar women, about 108 would have a sarcoma; MRI would flag about 292, of whom about 70 would have a sarcoma; and about 38 sarcomas would remain among the 99,708 with a negative scan.

Each of these assumptions may not hold. Women with a negative MRI had no baseline histology, and later sarcomas were ascertained through follow-up. Some may have been missed, and some may have been diagnosed only after follow-up ended; the count depends on the length and completeness of follow-up. Verification was differential, suspicion rested on T2-weighted morphology rather than on diffusion criteria, and 12 of the 17 sarcomas were low-grade endometrial stromal sarcomas, with only five leiomyosarcomas [25]. The reconstructed proportions are therefore not equivalent to sensitivity or specificity measured in histology-verified accuracy studies, and, given this histological composition, they cannot be translated into leiomyosarcoma-specific performance. With those reservations, the reconstruction points to less reassurance after a negative scan than either scenario predicts. It does not establish that MRI performs less well in consecutive populations; it illustrates how uncertain the transportability of pooled estimates to such populations remains. The pooled estimates were obtained in women whose masses already looked atypical. A population dominated by unremarkable fibroids would be expected to yield higher specificity and, for less conspicuous tumours, lower sensitivity.

Where baseline risk is high, a negative result does not make the risk small. At 1.01%, the value modelled for women aged 75–79 years [21], a negative MRI would leave approximately 0.08% on the published likelihood ratio or 0.18% on the derived scenario. Observed rates in women older than 60 years approach 2–3% [13]. At those values, the corresponding figures are approximately 0.16–0.25% and 0.36–0.55%, within or above the range of unconditional risk reported for younger surgical populations in Table 1.

A positive result at low baseline risk is usually a false positive. At 0.108%, a positive MRI gives a post-test probability of about 1.4% on the published likelihood ratio, or about 68 false positives per true positive (envelope 31–149). On the derived scenario, the figures are about 0.83% and 120. At 0.360%, the corresponding figures are about 20 and 36 (Figure 2b). On the reconstruction above, the consecutive cohort had about three false positives per true positive. Its reconstructed false-positive proportion, 0.22%, was substantially lower than the rates implied by the pooled specificity estimates (4.0% and 10.9%); that figure rests on the same unverified assumptions, so the contrast is indicative and is not a formal comparison of specificity. In less selected populations, pooled estimates may understate the value of a positive expert report and overstate the reassurance provided by a negative one.

Several qualifications limit how far the derivation should be pushed. The pooled inputs come from case-control and enriched cohorts in which spectrum effects can distort discrimination; these design features limit the transportability of the scenario calculations and motivate the reconstruction from the consecutive cohort above. Pooled estimates assume a dichotomised result, whereas radiologists issue graded impressions and defer. They conceal severe heterogeneity, with I^2^ reaching 95.8% for specificity in one synthesis [45] and 83.9% for the negative likelihood ratio in the other [50]. For a new setting, the relevant uncertainty is the prediction region, not the confidence region. The shaded envelopes in Figure 2 and the ranges in Table S1 are illustrative scenario envelopes, not formal confidence or credible intervals. For published likelihood ratios, they span the reported 95% confidence limits of each ratio; for the derived scenario, they span the extreme combinations of the marginal limits of sensitivity and specificity, ignoring their correlation. All of them treat the baseline risk as known, although its own uncertainty is wide, so the true uncertainty exceeds what the figure displays; nor do the envelopes capture the downward bias that partly post-assessment baseline values may introduce.

These limitations support reporting residual risk as an approximate range rather than as a single point estimate. Current consent documents quote population risks by age band [24], a reasonable default before imaging. Once an expert MRI has been reported, the available evidence can inform discussion of the direction and approximate magnitude of the change in risk and of the uncertainty that remains, preferably with reference to a population resembling the woman’s own. The scenario calculations presented here should not themselves be used to assign an individualised post-test probability.

## 7. Modality-Specific Consequences of a Missed Sarcoma

The same residual risk carries different consequences according to whether the planned treatment produces tissue, disrupts tissue, or leaves the lesion in place.

### 7.1. Excisional Treatment and Tissue Extraction

Myomectomy answers the diagnostic question within days for the lesions removed; a sarcoma in a myoma left in place is not covered by that answer. The diagnostic advantage lies in specimen availability and the timing of histopathological diagnosis; it should not be interpreted as evidence that myomectomy is oncologically safer or appropriate when sarcoma is meaningfully suspected. The weak point of excisional treatment is extraction. The oncological case against uncontained morcellation rests on a meta-analysis of four retrospective studies and 202 patients with unexpected leiomyosarcoma. Morcellation was associated with higher overall recurrence (62% versus 39%; OR 3.16, 95% CI 1.38–7.26), higher intra-abdominal recurrence (39% versus 9%; OR 4.11, 95% CI 1.92–8.81), and higher mortality (48% versus 29%; OR 2.42, 95% CI 1.19–4.92) [73]; the component studies were unadjusted. Population-level linkage points the same way: laparoscopic supracervical hysterectomy or myomectomy, used as a surrogate for uncontained power morcellation, was associated with higher disease-specific mortality in occult uterine sarcoma, particularly leiomyosarcoma, but not in occult endometrial carcinoma [74]. That analysis is registry-based, and its exposure groups were not randomly assigned either. In the integrated health system cohort, among women with stage I leiomyosarcoma, the adjusted risk ratio for death at one year after power or non-power morcellation was 5.12 (95% CI 1.33–19.76, *p* = 0.020); comparisons at three years were underpowered [16].

Other analyses have reached different conclusions. A systematic review of outcome studies found the data highly biased and of poor quality, with no reliable evidence that morcellation substantially results in tumour upstaging [75]. A registry analysis of 301 leiomyosarcomas applied competing-risks regression: morcellation of stage pT1 tumours was associated with a higher two-year cumulative incidence of local recurrence (49% versus 26%; subdistribution hazard ratio 2.11, 95% CI 1.41–3.16, *p* < 0.001), a lower cumulative incidence of distant metastases at five years (29% versus 54%; subdistribution hazard ratio 0.52, 95% CI 0.32–0.84, *p* = 0.008), and no difference in overall survival [76]. Several factors plausibly contribute, and they operate on all three datasets: morcellated cohorts are selected for apparently benign, lower-stage, smaller tumours in younger women; the definition of morcellation varies across studies; earlier series predate contained extraction; and ascertainment of local recurrence is more complete in women under close surveillance after an unexpected malignancy. A cautious interpretation is that uncontained morcellation of an occult leiomyosarcoma is associated with more local and intraperitoneal recurrence, that its association with overall survival is inconsistent, and that the effect size cannot be estimated with confidence from data in which the exposure was never randomly assigned.

Dissemination is not confined to malignancy. Morcellation of histologically benign leiomyoma can seed the peritoneum: a systematic review put the incidence of parasitic myoma after laparoscopic morcellation at 0.12% to 0.95% [77], while among 87 women who happened to undergo a second laparoscopy after uncontained power morcellation, parasitic myomas were found in 16 (18.4%), three-quarters of them asymptomatic [78]. The difference reflects ascertainment, symptom-triggered in the first and visual in the second.

Containment is the principal mitigation, but the evidence supporting it remains limited. A Cochrane review identified two trials and 176 premenopausal women, judged the evidence very low quality, and found no postoperative leiomyosarcoma in either group [79]; because no sarcoma occurred, whether containment prevents dissemination remains unanswered. Direct measurement is less reassuring. In a multicentre prospective series of 76 contained power morcellation procedures, dye or tissue was found outside the system in seven cases (9.2%), with the bag macroscopically intact in every one [80]. Sequential pelvic washings in 50 contained manual morcellations recovered myometrial cells in four women (8%, 95% CI 2–19%), and in most the cells were already present before morcellation began, which locates part of the spillage in the hysterectomy itself [81]. Neither endpoint measures failure of the containment system or malignant dissemination. Implementation is also incomplete. Among 67,115 laparoscopic supracervical hysterectomies in a US database, power morcellator use fell from 66.7% in late 2013 to 13.3% by mid-2018, containment bag use rose only from 5.2% to 15.2%. Bag use carried a higher perioperative complication rate (4.5% versus 3.3%; adjusted risk ratio 1.35, 95% CI 1.12–1.64) [82].

Restricting morcellation has had measurable costs. Among 25,571 women undergoing hysterectomy for fibroids in a national surgical quality programme, major complications rose from 1.9% to 2.4% after the FDA warning (adjusted OR 1.23, 95% CI 1.04–1.47, *p* = 0.020) and minor complications from 2.7% to 3.3% (adjusted OR 1.21, 95% CI 1.04–1.40, *p* = 0.010). Open surgery increased from 37.2% to 43.0% [83]. Myomectomy shifted similarly, from 49.1% abdominal to 60.0% [84]. The picture is not uniform. One large analysis found the route changed without a significant change in 30-day outcomes [85], and a national cost-effectiveness model judged the practice change cost-effective in its base case while noting that parameter uncertainty could alter the result [86]. These are before-and-after comparisons in which the warning was not the only thing that changed, so none should be read as a measured causal effect, and the balance of harm and benefit remains contested.

### 7.2. Embolic and Ablative Treatment: A Deferred Diagnosis

Embolisation and ablation change the problem qualitatively. In the EMMY trial, 35% of the 81 women treated by embolisation had undergone hysterectomy by 10 years, 24 of 77 (31%) even after a technically successful procedure [87]; roughly two-thirds therefore did not provide a specimen within 10 years. When an occult sarcoma is present, persistent or recurrent symptoms, interval growth, atypical imaging evolution, or apparent treatment failure may prompt reassessment; the interval to diagnosis therefore depends partly on how and when such findings are investigated. Two published cases mark the range: continued growth of fibroids in a postmenopausal woman leading to a hysterectomy nearly fatal from venous bleeding [88], and an endometrial stromal sarcoma identified six months after embolisation [89]. Case reports illustrate that this sequence can occur; they cannot establish its frequency.

Ablative techniques share this structure and add a second problem. Two routes should be distinguished, because they differ in what the operator can see. In its pivotal single-arm trial, transcervical RFA achieved a 50% or greater reduction in menstrual bleeding in 64.8% of women (95% CI 56.3–72.6%), with 99.3% (95% CI 95.1–99.9%) free from surgical reintervention at 12 months [90]. Pooled prospective data across 1283 patients, combining laparoscopic, transvaginal, and transcervical routes, gave cumulative reintervention rates of 4.2%, 8.2%, and 11.5% through three years [91]; an independent retrospective series reported 45.3% of 53 women undergoing reintervention and 13.2% eventual hysterectomy [92]. None of these treatment datasets provides histological assessment from the index ablation procedure, and the laparoscopic route at least permits inspection of the serosa where the transcervical route does not. A second concern is that prior ablation may alter the morphology of a subsequently resected specimen and complicate histopathological interpretation; in one reported case, a hysterectomy specimen obtained after radiofrequency ablation was initially classified as epithelioid leiomyosarcoma and reclassified as benign only on review [39]. Diagnostic misclassification after ablation can therefore occur in both directions.

The largest denominator comes from ultrasound-guided focused ultrasound, in the cohort from which the exploratory reconstruction in Section 6 was derived: 17 sarcomas among 15,759 women (0.108%), 12 of them low-grade endometrial stromal sarcomas; six were diagnosed only after ablation, the pretreatment MRI having raised no suspicion [25]. Among the six women operated on after ablation, the authors reported no histological dissemination of the sarcoma, a number too small to exclude the risk [25]. A leiomyosarcoma has also been reported five years after magnetic resonance-guided focused ultrasound, a related but distinct technique with its own screening requirement [93]. In specimen-free treatment, the principal diagnostic concern considered here is delayed recognition: a missed sarcoma may remain unrecognised until later clinical, imaging, or pathological reassessment. Two limits belong with that argument. Its evidential base is three case reports and one large cohort whose authors reported no histological dissemination among the six women operated on after ablation, so the position is structural rather than demonstrated. And it has no stated comparator: the realistic alternative for a woman declining hysterectomy is usually uterus-preserving surgery, which carries its own risk of mechanical dissemination, so it does not follow that a woman with an occult sarcoma treated by ablation fares worse than one treated by morcellated excision.

### 7.3. Pharmacological Suppression and the Presenting Signal

**Figure 3:**
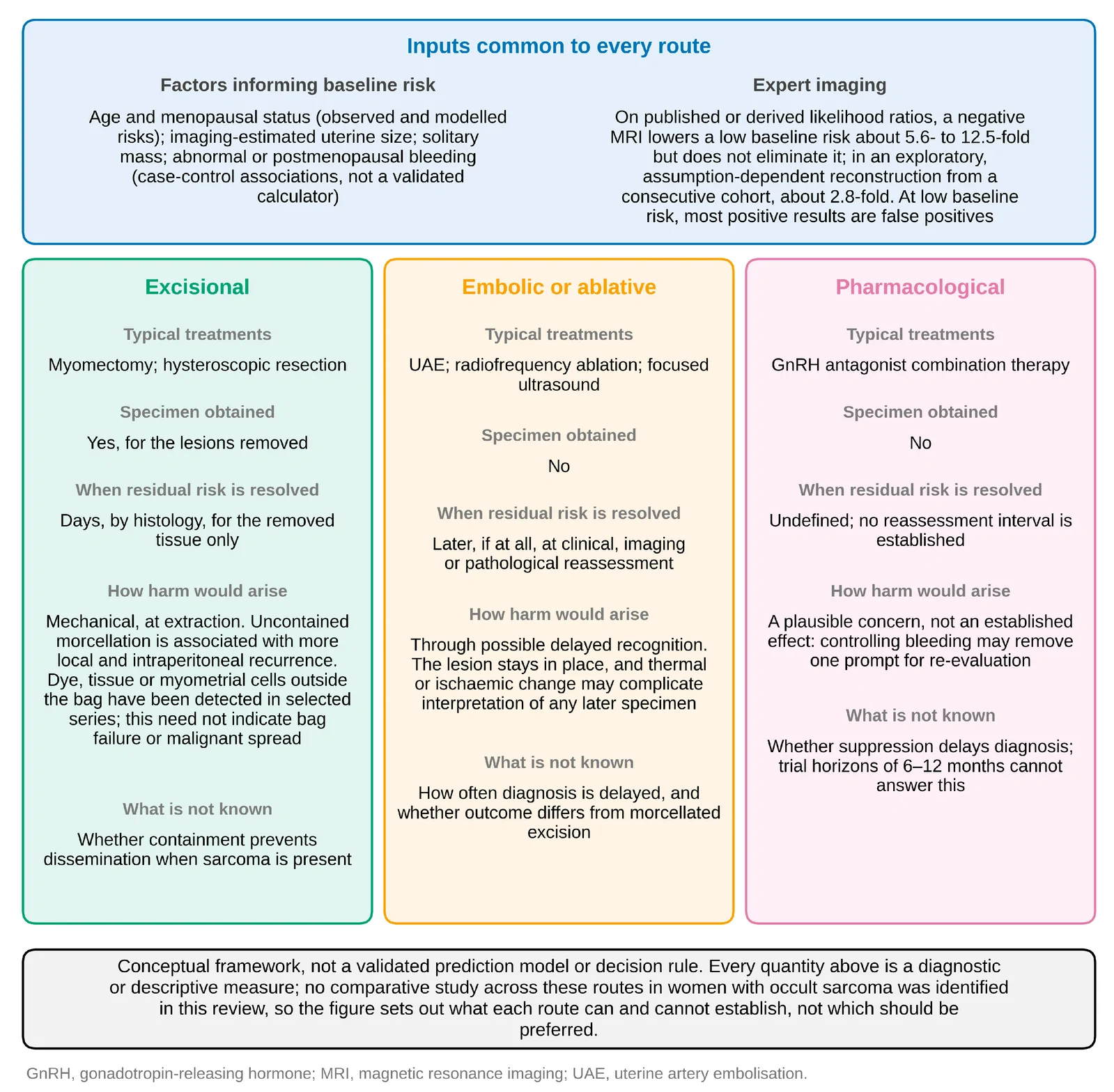
Conceptual framework for characterising residual diagnostic uncertainty according to uterus-preserving treatment type. The upper panel gives the factors informing baseline risk and the contribution of expert imaging, drawn from Section 3 and Section 6. The three columns set out, for each route, specimen status, when a residual risk would be resolved, the mechanism by which harm could arise if sarcoma were present, and what remains unknown. Specimen availability is a diagnostic distinction: it concerns when histopathological resolution may become available, does not establish comparative oncological safety, and does not imply that myomectomy is oncologically preferable when sarcoma is meaningfully suspected. The figure is non-directional and makes no comparative treatment recommendation: it contains no decision node, threshold or recommended order, because no comparative study across these routes in women with occult sarcoma was identified in this review. Values are those reported in Section 5, Section 6 and Section 7 [25,39,73,77,80,81,94]. The consecutive-cohort value in the upper panel is an assumption-dependent reconstruction, not a diagnostic-accuracy estimate (Section 6). GnRH, gonadotropin-releasing hormone; MRI, magnetic resonance imaging; UAE, uterine artery embolisation.

Gonadotropin-releasing hormone antagonist combination therapy is well supported. Relugolix combination therapy produced a response in 73% and 71% of women across two replicate phase 3 trials, against 19% and 15% with placebo (*p* < 0.001 for both) [94], and elagolix with add-back achieved 68.5% and 76.5% versus 8.7% and 10% [95]. These primary endpoints all measure bleeding. Relugolix reduced uterine volume, but not fibroid volume [94], a reminder that symptomatic response does not establish the nature of the lesion, and that volumetric change is itself a surrogate rather than a patient-important outcome. Figure 3 synthesises these three treatment routes according to specimen availability, the potential timing of histopathological confirmation, the consequences of a missed sarcoma, and the remaining uncertainties.

No study retrieved for this review has tested whether medical therapy delays the diagnosis of uterine sarcoma, and the available evidence does not support such an assertion. The concern is structural rather than evidenced, and its weakness should be visible: the bleeding pattern most strongly associated with sarcoma in case-control data is postmenopausal bleeding [22], whereas the phase 3 antagonist trials enrolled premenopausal women with heavy menstrual bleeding, so the marker does not transfer directly to the treated population. Medical therapy can also generate a signal: UK consent advice treats failure of fibroids to shrink under gonadotropin-releasing hormone agonist treatment as a red flag in premenopausal women [24]. What can be said is narrower. A treatment whose principal effect is to control the presenting symptom may remove one signal that would otherwise prompt re-evaluation, and whether this translates into later-stage diagnosis is a question that trial datasets with 6- to 12-month horizons cannot answer.

## 8. Convergence and Divergence Among Guidance Documents

The major guidance documents examined here converge on the central factual premise. The American College of Obstetricians and Gynecologists states that leiomyosarcoma cannot be reliably diagnosed preoperatively, that morcellation of a malignancy is contraindicated, and that management should follow shared decision-making [96]; the Society of Gynecologic Oncology similarly records that no reliable method exists to distinguish benign from malignant smooth muscle tumours before removal [97]. Regulators also converge on the age criterion. The FDA recommends laparoscopic power morcellation only with a legally marketed containment system, not when malignancy is known or suspected, and not in women over 50 years of age or postmenopausal women, a restriction that its labelling guidance frames as a contraindication [7]. The National Institute for Health and Care Excellence states that the procedure should not be used in people who are postmenopausal or over 50 and, below that threshold, only with special arrangements for clinical governance, consent, and audit or research [8]. Both restrictions apply to laparoscopic power morcellation as a method of tissue extraction; they should not be read as contraindications to myomectomy itself or to uterine artery embolisation, radiofrequency ablation, focused ultrasound or other uterus-preserving treatment. UK consent advice asks clinicians to discuss the risk of unsuspected sarcoma and lists red-flag features [24].

The documents diverge on tissue. European guidance prefers preoperative pelvic MRI, or transvaginal or transabdominal ultrasound by an expert sonographer at a highly specialised site, with interpretation by subspecialised radiologists. For the most suspicious lesion, it recommends ultrasound-guided trans-uterine core-needle biopsy of 14–16 gauge or larger with expert pathological review (grade B). It also advises that morcellation always be avoided where sarcoma is suspected, and that unexpected sarcoma after myomectomy be followed by hysterectomy [98,99]. North American statements do not recommend directed biopsy of the myometrial mass, and endometrial sampling continues to serve as the default tissue test despite naming the correct histological type in only 64% of sampled uterine sarcomas [64]. Because the published biopsy series are small [65,66], this reflects a genuine evidential gap rather than a simple disagreement. The limited yield of endometrial sampling for sarcoma leaves its wider role unaffected. The American College of Obstetricians and Gynecologists observes that endometrial adenocarcinoma most often presents with abnormal uterine bleeding or postmenopausal bleeding and advises appropriate preoperative evaluation so that a coexisting endometrial malignancy is not missed [96]; UK consent advice asks for an endometrial biopsy where indicated [24].

The gynaecological documents examined here are organised mainly around morcellation: what may be morcellated, by whom, in what container and after what counselling [7,8,96,97,100]. UK consent advice is a partial exception: it notes that any uterus-preserving option, including embolisation and medical management, runs the risk of leaving an undiagnosed sarcoma in situ, and that neither ultrasound nor MRI can definitively exclude sarcomatous change [24]. A supplementary search of interventional radiology and device guidance shows that specimen-free treatments are not unregulated in this respect. European standards of practice for uterine artery embolisation recommend pretreatment imaging, preferably contrast-enhanced MRI, to confirm the diagnosis and exclude other pathology, and list uterine malignancy as an absolute contraindication [101]. Manufacturer labelling for a transcervical radiofrequency ablation system lists known or suspected gynaecological malignancy as a contraindication; this is device labelling rather than professional guidance [102]. These documents address suspected malignancy qualitatively, as a reason not to treat. Within the documents examined, we did not identify guidance on how the residual risk after a reassuring examination should be estimated or communicated. Nor did we identify guidance on how a sarcoma missed before specimen-free treatment should be sought afterwards. The supplementary search was targeted rather than comprehensive. The gynaecological documents endorse counselling about the risk of unsuspected sarcoma, and the European guidance makes this an explicit graded recommendation [98,99]. Table 3 summarises the positions of regulatory agencies, professional societies, and device labelling on pretreatment assessment, age-related restrictions, and morcellation.

## 9. Discussion

### 9.1. What the Evidence Supports

Four conclusions can be stated with reasonable confidence. Occult uterine sarcoma is uncommon in women treated for presumed fibroids, but reported frequencies differ between clinical populations. Most contemporary series lie between about 0.1% and 0.4% for all sarcoma; leiomyosarcoma alone is less frequent, and observed rates for all sarcoma approach 2–3% after the age of 60 years [13,15,16,17,21,25]. Age is the most consistently reported risk modifier available before treatment, although no validated individual risk calculator exists [13,21,22]. Separate MRI syntheses report pooled sensitivity of 84–90% and specificity of 89–96%, although their pathological target conditions differ and the more recent synthesis included heterogeneous malignant cohorts beyond uterine sarcoma; these estimates come largely from retrospective and enriched series [45,50]. These estimates are higher than those reported for ultrasound in a different set of studies [47]. Uncontained morcellation of an occult leiomyosarcoma is associated with more local and intraperitoneal recurrence [73,76].

Several conclusions warrant qualification. MRI does not provide absolute exclusion of sarcoma. On published or derived likelihood ratios, a negative study lowers the odds about 5.6- to 12.5-fold; in women older than 60 years, the residual can remain within or above the untested risk of younger surgical populations. These likelihood-ratio figures are population-level scenarios for all uterine sarcoma: because their baseline risks are partly post-assessment quantities, they may understate residual risk, and they are not validated individual probabilities. In the exploratory reconstruction from the consecutive cohort, made under unverified assumptions, the corresponding reduction was about 2.8-fold and the proportion corresponding to sensitivity was 64.7%; these quantities concern all uterine sarcoma, are not independently observed diagnostic-accuracy estimates, and should not be compared directly with the pooled estimates given above [25]. Evidence relating morcellation to recurrence is more consistent than evidence for an effect on overall survival [76]. Reported serum biomarker performance remains heterogeneous, with LDH sensitivity ranging from 0.47 to 1.00 across studies [57]. For directed biopsy, the difficulty is of a different kind: the published series are small and selected, so the evidence is insufficient to establish diagnostic accuracy and clinical utility in unselected populations, which is not evidence that the technique is ineffective [65,66]. The performance figures in Section 5 and Section 6 are diagnostic surrogates: no study cited here shows that a pretreatment test, threshold, or stratification improves survival, recurrence, stage at diagnosis or other patient-important outcomes.

Clinical interpretation of a residual probability should take account of the consequences of diagnostic misclassification in the context of the planned treatment. When myomectomy is planned, histopathological examination can resolve the diagnostic question for the removed tissue within days, although not for lesions left in the uterus, and the operative question becomes extraction technique. When embolisation or ablation is planned, the same residual risk persists after treatment and may remain unresolved until subsequent clinical, imaging, or pathological reassessment. When medical suppression is planned, it is plausible, though unproven, that controlling the presenting symptom removes one prompt for reassessment. This is a conceptual framework for counselling, not a validated decision rule.

### 9.2. Research Priorities

Several evidence gaps remain. We did not identify a diagnostic-accuracy study of MRI conducted in a consecutive, unselected cohort with complete verification. The nearest approach, in which negatives were verified only by follow-up, permits no more than an assumption-dependent reconstruction, which cannot be compared directly with pooled accuracy estimates [25]. A high-priority research need is a properly verified consecutive cohort, with diagnostic accuracy reported separately for leiomyosarcoma and other sarcoma subtypes. ADC thresholds range from 0.87 to 1.29 × 10^−3^ mm^2^/s without demonstrable differences in performance [42], and acquisition parameters are not standardised, so a harmonised protocol is a prerequisite for pooling; future syntheses should also report estimates from joint models, with prediction regions [72]. Prospective external validation of composite scores would provide stronger evidence than additional model refinement within case-control samples [49,59,60]. Prospective registries of women treated by embolisation, ablation and focused ultrasound, with five-year minimum follow-up and mandatory reporting of subsequent malignancy, would extend the published case reports illustrating delayed recognition after specimen-free treatment [89,93]. Directed core-needle biopsy would benefit from multicentre evaluation in less selected populations, powered to characterise the false-negative rate [65,66], and cell-free nucleic acid classifiers require validation cohorts that include benign leiomyoma with detectable copy-number alterations [61,62]. We did not identify a study comparing outcomes in women with occult sarcoma treated by ablative or embolic therapy with outcomes after morcellated excision.

### 9.3. Strengths, Limitations, and Contribution

The principal limitations arise from both the underlying evidence and the design of this narrative review. The central limitation of the quantitative section is the baseline risk. The values used in Section 6 are representative population-level scenarios for all uterine sarcoma. Most are partly post-assessment quantities and not true pre-imaging probabilities, and each is itself uncertain. Applying a further imaging likelihood ratio to them may underestimate residual risk, because prior assessment and the subsequent test are unlikely to be conditionally independent. The resulting figures are therefore neither validated individual probabilities nor leiomyosarcoma-specific ones. Most diagnostic accuracy estimates derive from case-control or two-gate assembly, in which a sarcoma group drawn from referral or registry sources is compared with a separately collected leiomyoma group; such assembly can distort apparent discrimination through spectrum and selection effects and limits the transportability of predictive values to unselected clinical populations, as the exploratory, assumption-dependent reconstruction in Section 6 illustrates. Heterogeneity is substantial and not fully explained, with I^2^ reaching 95.8% for pooled specificity [45] and apparent diffusion coefficient thresholds varying by half their own magnitude [42]. Target definitions vary across studies: “sarcoma” variously includes or excludes endometrial stromal sarcoma and adenosarcoma and, in some diagnostic cohorts, extends to STUMP or carcinosarcoma [45,56], “morcellation” spans power, non-power and scalpel techniques, and STUMP is defined by criteria that differ between series [35]. The scenario calculations also combine likelihood ratios from univariate syntheses, and their envelopes have no defined coverage. Generalisability is limited in a specific direction, since the prevalence literature is built largely on hysterectomy cohorts older than the women who present for uterus-preserving treatment. Disaggregation by race is rare, although one population-based cohort reported occult sarcoma in 0.28% of African-American, 0.36% of White and Hispanic, and 0.44% of Asian and other women [13,16], despite the higher burden of fibroid disease in Black women [1]. Follow-up in the ablative literature is routinely shorter than the interval over which a missed sarcoma would declare itself. Publication and selective-reporting bias cannot be excluded. The concern is greatest for small diagnostic, biomarker, and composite-score studies reporting very high discrimination or near-perfect sensitivity or specificity, because favourable findings may be more likely to be published and performance metrics may be reported selectively. Exceptionally high estimates from small or selected cohorts should therefore be regarded as hypothesis-generating until reproduced in independent, clinically representative populations. In keeping with the narrative design, no formal assessment of such bias was undertaken. This review also has methodological limitations: the search was purposive rather than exhaustive, was confined to English-language publications, and concentrated on morcellation-related guidance, with interventional radiology and device documents identified through a targeted supplementary search.

Strengths of this review include the auditability of the quantitative framework, explicit presentation of discordant evidence, and an exploratory reconstruction from a consecutive cohort, under stated assumptions, set beside the pooled diagnostic estimates. The quantitative core is auditable: every post-test probability follows by a stated rule from published likelihood ratios, or from pooled sensitivity and specificity where likelihood ratios were not reported, with every input and output tabulated (Table S1). Discordant estimates, including two MRI meta-analyses and three morcellation-outcome datasets that point in different directions, are presented alongside their populations and methods rather than combined into a single summary interpretation. The reconstruction from a consecutive cohort, under stated and unverified assumptions, illustrates that the pooled estimates may not transfer unchanged outside the enriched series in which they were obtained; it does not quantify the difference [25].

The contribution is deliberately limited. The review brings tumour biology, test performance and prevalence into a single frame, so that the residual risk after a negative test is expressed in the absolute terms in which consent is obtained rather than left implicit in sensitivity figures. It also organises the question by whether the intended treatment yields a specimen, a distinction that is not consistently made explicit in the guidance documents examined and that influences whether a missed diagnosis is likely to be recognised within days or only after years.

## 10. Conclusions

Occult uterine sarcoma is uncommon among women treated for presumed fibroids, but it is clinically consequential, and its baseline risk differs substantially between clinical populations and rises with age. Imaging, particularly expert MRI with diffusion-weighted sequences, contributes meaningful diagnostic information but does not reliably exclude sarcoma, and post-test risk estimates depend strongly on how the underlying diagnostic studies selected their patients and on the population to which they are applied. For directed biopsy, the evidence is insufficient to establish diagnostic accuracy in unselected populations, which is not the same as evidence of ineffectiveness. No currently available test, alone or in combination, eliminates diagnostic uncertainty.

The consequences of that uncertainty depend on the treatment. When the treated lesion is excised, histopathological examination can resolve the diagnostic question for the removed tissue within days; lesions left in the uterus remain uncharacterised. After embolisation, ablation or medical therapy, no histological diagnosis is obtained at treatment, and a missed sarcoma may remain unrecognised until later reassessment, which makes specimen-free treatment a distinct diagnostic problem.

Prospective consecutive diagnostic cohorts with complete verification and separate reporting for leiomyosarcoma, and long-term registries of women treated without a specimen, are needed. The framework proposed here, which considers absolute residual risk together with the specimen status of the intended treatment, is conceptual and requires prospective validation before it could be used as a clinical decision tool.

## Figures and Tables

**Table 1:** Reported frequency of uterine sarcoma or leiomyosarcoma in women treated for presumed leiomyoma.

Source	Design and Setting	Population	Principal Estimate	Principal Limitation
Parker et al., 1994 [23]	Retrospective review, two community hospitals, 1988–1992	1332 operations for presumed leiomyoma, 371 for rapid growth	Uterine sarcoma 0.23%; no sarcoma among 198 women meeting a published definition of rapid growth	Predates contemporary imaging; low power to exclude a small effect
Pritts et al., 2015 [15]	Bayesian binomial random-effects meta-analysis, 133 studies	Surgery for presumed fibroids	LMS 0.51 per 1000 (95% CrI 0.16–0.98); prospective studies only 0.12 per 1000 (95% CrI < 0.01–0.75)	Heterogeneous inclusion; most component studies retrospective
Brohl et al., 2015 [21]	Retrospective cohort with pooled analysis and age modelling	2075 myomectomies; pooled with eight published series	Uterine sarcoma 2.94 per 1000 (95% CI 1.8–4.1), the sum of LMS 1.78 per 1000 (10,120 patients), and other sarcoma 1.16 per 1000 (6889 patients); predicted 10.1 per 1000 at age 75–79 years	Age-specific values are modelled rather than directly observed
Lieng et al., 2015 [18]	Retrospective cohort, single department, 2000–2013	4791 women referred with anticipated benign fibroids	26 LMS among 4791 women (0.54%), most suspected preoperatively; six occult (0.13%) in the regulatory re-analysis [13]; unintended morcellation of an LMS in one woman	Single centre; estimate strongly shaped by preoperative triage
Raine-Bennett et al., 2016 [16]	Population-based cohort, integrated health system, 2006–2013	34,728 hysterectomies for leiomyomas	Sarcoma 3.60 per 1000 (95% CI 2.97–4.23), 1 in 278; LMS 2.33 per 1000 (95% CI 1.83–2.84), 1 in 429	Hysterectomy cohort, older than typical candidates for uterus-preserving treatment
US Food and Drug Administration, 2017 [13]	Regulatory quantitative assessment, 23 studies published 2014–2017	Surgery for uterine fibroids	Sarcoma 0.277% (95% CI 0.172–0.445%); LMS 0.134% (95% CI 0.089–0.202%), random-effects estimates	Overlapping source studies; not peer-reviewed
Cao et al., 2019 [20]	Retrospective cohort, 2009–2016	26,643 patients operated on for presumed leiomyoma	Unexpected uterine sarcoma 0.33% (29 LMS, 48 endometrial stromal sarcoma, 11 adenosarcoma)	Retrospective ascertainment; single country
Multinu et al., 2019 [17]	Population-based cohort, 1999–2013	3759 hysterectomies for benign indication	Unexpected sarcoma 0.13% (95% CI 0.04–0.31%); 0.39% among operations for fibroids; 0.35% premenopausal versus 0.57% peri- or postmenopausal	Five events overall; subgroup estimates rest on one to four cases
Wang et al., 2021 [25]	Retrospective consecutive cohort presenting for HIFU, 2008–2019	15,759 women, all with pretreatment MRI; later extended to 41,631 women in 13 hospitals [26]	Uterine sarcoma 0.108% (17 of 15,759; 12 low-grade ESS, 5 LMS); six diagnosed only after ablation	Ascertainment of occult cases depends on completeness of follow-up
Chantasartrassamee et al., 2022 [22]	Retrospective case-control within a surgical cohort	18,218 women operated on for a uterine mass	Uterine sarcoma 0.37% (68 of 18,218)	Single institution; case-control comparison nested within the cohort
Zouzoulas et al., 2023 [19]	Retrospective single-centre series, 2011–2018	803 operations for uterine fibroids	LMS 0.75% (six of 803), all six suspected preoperatively, so none occult and none morcellated; five ESS (0.65%)	Referral centre; small numerator

CI, confidence interval; CrI, credible interval; ESS, endometrial stromal sarcoma; HIFU, high-intensity focused ultrasound; LMS, uterine leiomyosarcoma; MRI, magnetic resonance imaging. Estimates are reported as given in the source. Rows are ordered by year of publication. Most estimates describe sarcoma unsuspected after routine preoperative assessment; where suspected cases are included, this is stated. The condition counted is given for each estimate as reported by its source: uterine sarcoma as a group, LMS alone or, where stated, another subtype. These are not interchangeable, and only all-sarcoma estimates are carried into the scenarios of Section 6. One of them, the 0.39% estimate among hysterectomies for fibroids [17], is reported by its source as unexpected sarcoma, although all four events were LMS. Estimates of sarcoma unsuspected after preoperative assessment are partly post-assessment quantities and not true pre-imaging probabilities.

**Table 2:** Pooled and validation-level performance of pretreatment tests for discriminating uterine sarcoma from leiomyoma. Study-level detail for the individual imaging, biomarker and biopsy studies is given in Table S2.

Test and Source	Target Condition Reported by Source	Design; Population	Sensitivity; Specificity (95% CI)	Other Reported Metrics	Principal Limitation
Ultrasound, meta-analysis [47]	All uterine sarcoma	Meta-analysis; 972 women (694 leiomyoma, 278 sarcoma)	0.76 (0.70–0.81); 0.89 (0.87–0.92)	LR+ 6.65 (4.45–9.93); LR− 0.26 (0.07–1.00); AUC 0.8925	LRs pooled separately from sensitivity and specificity; wide LR− interval reflects between-study heterogeneity
MRI, meta-analysis [50]	All uterine sarcoma	Eight studies; 2495 women	0.90 (0.84–0.94); 0.96 (0.96–0.97)	LR+ 13.55 (6.20–29.61); LR− 0.08 (0.02–0.32); AUC 0.9759	LRs pooled separately and not derivable from the pooled sensitivity and specificity; I^2^ 79.6% (sensitivity) and 71.8% (specificity)
MRI, meta-analysis [45]	Heterogeneous malignant target; contributing cohorts included sarcoma, STUMP, carcinosarcoma and adenosarcoma	Twelve retrospective studies, 2019–2025; masses suspected of sarcoma	84.2% (76.3–89.9%); 89.1% (78.1–94.9%)	AUC 0.916 (0.876–0.941)	I^2^ 66.3% for sensitivity and 95.8% for specificity
ADC measurement, meta-analysis [42]	All uterine sarcoma	21 studies	89% (82–94%); 86% (78–92%)	AUC 0.94 (0.92–0.96); thresholds 0.87–1.29 × 10^−3^ mm^2^/s; pooled means 0.904 versus 1.287 × 10^−3^ mm^2^/s	Specificity 82% (75–89%) when ADC is read alone
Circulating biomarkers, meta-analysis [57]	All uterine sarcoma	20 observational studies; 13 biomarkers	LDH 0.47–1.00; CA-125 0.43–0.64; CRP 0.84–0.87; LDH 0.86–1.00; CA-125 0.50–0.77; CRP 0.83–0.89	Ranges across studies rather than pooled estimates	Marked between-study heterogeneity; no single usable threshold
DWI-based algorithm, derivation and external validation [43]	Malignant tumours, including STUMP and carcinosarcoma	Case-control; training 51 sarcomas and 105 leiomyomas plus two validation sets	98% (90–100%) training; 83% (65–92%) less-experienced reader; 94% (88–98%); 97% (83–99%)	ADC threshold 0.905 × 10^−3^ mm^2^/s	Case-control assembly; sensitivity falls with reader inexperience
Consensus MRI algorithm, external validation [53]	Heterogeneous uterine malignancy, as defined by the source (LMS, other sarcomas and carcinosarcoma)	40 pelvic MRI examinations, two blinded readers	75% and 81.2%; 95.8% and 91.7%	κ 0.89; sensitivity and NPV 100% when restricted to LMS	16 malignancies; single centre
pLMS composite score, external validation [60]	Leiomyosarcoma only	90 new leiomyosarcomas and 659 prospectively recruited leiomyomas	Not reported	AUC 0.97; 0.960 versus 0.845 in head-to-head comparison	Case-control assembly may overestimate apparent discrimination because of spectrum and design effects and limits transportability to unselected clinical populations
Endometrial sampling [64]	All uterine sarcoma	730 patients with reviewable sampling; 72 of 142 uterine sarcomas sampled	Not estimable; the correct histological type was identified in 46 of all 142 sarcomas; Not reported	Invasive tumour identified in 86% (62 of 72) and the correct histological type in 64% (46 of 72) of sampled sarcomas, versus 81% for epithelial tumours (*p* < 0.001)	Conditional on a sample having been obtained; sarcoma as a group, with no LMS-specific denominator

ADC, apparent diffusion coefficient; AUC, area under the receiver operating characteristic curve; CA-125, cancer antigen 125; CI, confidence interval; CRP, C-reactive protein; DWI, diffusion-weighted imaging; LDH, lactate dehydrogenase; LMS, uterine leiomyosarcoma; LR+, positive likelihood ratio; LR−, negative likelihood ratio; MRI, magnetic resonance imaging; NPV, negative predictive value; STUMP, smooth muscle tumour of uncertain malignant potential. Target conditions are those reported by each source. “All uterine sarcoma” denotes that the source treated uterine sarcoma as a single target without restriction to LMS; such estimates are not LMS-specific, and estimates for different target conditions are not interchangeable. The first five entries are evidence syntheses.

**Table 3:** Positions of regulatory agencies, professional societies and device labelling on morcellation and pretreatment assessment.

Body and Document	Position on Morcellation	Age or Menopausal Restriction	Pretreatment Assessment Specified
US Food and Drug Administration, safety communication, 2020 [7]	Only with a legally marketed containment system; not to be used when malignancy is known or suspected	Not to be used in women older than 50 years or postmenopausal; a contraindication in labelling guidance	Thorough preoperative screening; content not specified
National Institute for Health and Care Excellence, HTG589 (formerly IPG703), 2021 [8]	Aged 50 or under or premenopausal: only with special arrangements for clinical governance, consent, and audit or research	Should not be used in people older than 50 years or postmenopausal	Not specified
Royal College of Obstetricians and Gynaecologists, Consent Advice No. 13, 2019 (updated June 2024) [24]	Consent must cover the risk of unsuspected sarcoma	Morcellation should not be recommended in individuals who are postmenopausal or over 50 years of age	Red-flag review, including suspicious imaging features and, if indicated, endometrial biopsy; ultrasound or MRI before morcellation, neither of which definitively excludes sarcoma
American College of Obstetricians and Gynecologists, Committee Opinion 822, 2021 [96]	Morcellation of a malignancy is contraindicated; leiomyosarcoma cannot be reliably diagnosed preoperatively	No absolute age threshold; shared decision-making required	Evaluation for increased risk of corpus malignancy
Society of Gynecologic Oncology, position statement [97]	Generally contraindicated with documented or highly suspected malignancy; contained extraction listed as an alternative	Not specified	States that no reliable preoperative discrimination exists
ESGO, EURACAN and GCIG, 2024 guideline and 2025 pocket guidelines [98,99]	Always to be avoided where sarcoma is suspected (Grade A); counselling on the low risk of unsuspected sarcoma required (Grade A)	Not framed as an age threshold	Pelvic MRI preferred, or expert ultrasound at a specialised site (Grade B); subspecialised radiological interpretation (Grade A); ultrasound-guided trans-uterine core-needle biopsy (≥14–16 G) of the most suspicious lesion with expert pathological review (Grade B)
CIRSE, Standards of Practice: uterine artery embolisation, 2015 [101]	Not applicable; no tissue is extracted	Not specified in the sections examined	Ultrasound or MRI to confirm leiomyoma and exclude other pathology; contrast-enhanced MRI preferred (level 2); uterine or ovarian malignancy an absolute contraindication
Transcervical RFA system, manufacturer labelling (safety information), 2025 [102]	Not applicable; no tissue is extracted	Not specified in the document examined	Known or suspected gynaecological malignancy listed as a contraindication

CIRSE, Cardiovascular and Interventional Radiological Society of Europe; ESGO, European Society of Gynaecological Oncology; EURACAN, European Reference Network on Rare Adult Solid Cancers; G, gauge; GCIG, Gynecologic Cancer InterGroup; MRI, magnetic resonance imaging; RFA, radiofrequency ablation. Grades of recommendation are those assigned in the source documents; the CIRSE level of evidence is given as stated there. Interventional radiology and device documents were identified by a supplementary targeted search and do not form a comprehensive inventory. The age and menopausal restrictions in the first two rows apply to laparoscopic power morcellation, not to myomectomy itself or to other uterus-preserving treatment.

## Data Availability

No new data were created or analysed in this study. Data sharing is not applicable to this article.

## Supplementary Materials

The following supporting information can be downloaded at: https://www.mdpi.com/article/10.3390/life16101738/s1, Table S1: Illustrative post-test probability of uterine sarcoma across representative baseline-risk scenarios, with all inputs; Table S2: Study-level performance of the individual imaging, biomarker, score and biopsy studies; Table S3: Location within the manuscript of the elements addressed by the SANRA criteria.
