# Supplementary material for: Pretreatment Assessment of Occult Uterine Sarcoma Risk Before Uterus-Preserving Treatment of Presumed Fibroids: A Narrative Review
Source: Life (Basel). 2026 Oct 16;16(10):1738. doi: 10.3390/life16101738 (PMC13706578; doi:10.3390/life16101738)
Supplement: Supplementary file 1 [file life-16-01738-s001.zip › life-4614120-supplementary.pdf]

Supplementary Materials

Pretreatment Assessment of Occult Uterine Sarcoma Risk Before Uterus-Preserving Treatment of Presumed Fibroids: A Narrative Review

Reference numbers correspond to the reference list of the main manuscript.

Table S1. Illustrative post-test probability of uterine sarcoma across representative baseline-risk scenarios. Baseline-risk scenarios refer to all uterine sarcoma. These are population-level scenario calculations. They are not individualised risk estimates, and the table is neither a validated prediction model nor a set of clinical decision thresholds.

Baseline-risk scenario and source	Test and likelihood ratios used	After a negative result: post-test probability (scenario envelope); 1 in N	After a positive result: post-test probability (scenario envelope)	False-positive results per true positive (envelope)
0.108% (1 in 926); consecutive cohort presenting for focused-ultrasound ablation [25]	MRI; published pooled LR+ 13.55, LR− 0.08 [50]	0.0086% (0.0022–0.035%); 1 in 11,563	1.4% (0.67–3.1%)	68 (31–149)
	MRI; derived LR+ 7.72, LR− 0.177, secondary scenario [45]	0.019% (0.012–0.033%); 1 in 5,217	0.83% (0.38–1.9%)	120 (52–265)
	Ultrasound; published pooled LR+ 6.65, LR− 0.26 [47]	0.028% (0.0076–0.11%); 1 in 3,558	0.71% (0.48–1.1%)	139 (93–208)
0.13% (1 in 769); hysterectomy for benign indication [17]	MRI; published pooled LR+ 13.55, LR− 0.08 [50]	0.010% (0.0026–0.042%); 1 in 9,604	1.7% (0.80–3.7%)	57 (26–124)
	MRI; derived LR+ 7.72, LR− 0.177, secondary scenario [45]	0.023% (0.014–0.039%); 1 in 4,333	1.0% (0.45–2.2%)	99 (44–221)
	Ultrasound; published pooled LR+ 6.65, LR− 0.26 [47]	0.034% (0.0091–0.13%); 1 in 2,956	0.86% (0.58–1.3%)	116 (77–173)
0.277% (1 in 361); regulatory random-effects estimate [13]	MRI; published pooled LR+ 13.55, LR− 0.08 [50]	0.022% (0.0056–0.089%); 1 in 4,501	3.6% (1.7–7.6%)	27 (12–58)
	MRI; derived LR+ 7.72, LR− 0.177, secondary scenario [45]	0.049% (0.030–0.084%); 1 in 2,031	2.1% (0.96–4.7%)	47 (20–103)
	Ultrasound; published pooled LR+ 6.65, LR− 0.26 [47]	0.072% (0.019–0.28%); 1 in 1,386	1.8% (1.2–2.7%)	54 (36–81)
0.360% (1 in 278); population-based, hysterectomy for leiomyomas [16]	MRI; published pooled LR+ 13.55, LR− 0.08 [50]	0.029% (0.0072–0.12%); 1 in 3,461	4.7% (2.2–9.7%)	20 (9–45)
	MRI; derived LR+ 7.72, LR− 0.177, secondary	0.064% (0.038–0.11%); 1 in 1,562	2.7% (1.2–6.0%)	36 (16–79)

	scenario [45]			
	Ultrasound; published pooled LR+ 6.65, LR− 0.26 [47]	0.094% (0.025–0.36%); 1 in 1,066	2.3% (1.6–3.5%)	42 (28–62)
0.39% (1 in 256); surgery for uterine fibroids [17]	MRI; published pooled LR+ 13.55, LR− 0.08 [50]	0.031% (0.0078–0.13%); 1 in 3,194	5.0% (2.4–10%)	19 (9–41)
	MRI; derived LR+ 7.72, LR− 0.177, secondary scenario [45]	0.069% (0.042–0.12%); 1 in 1,441	2.9% (1.3–6.5%)	33 (14–73)
	Ultrasound; published pooled LR+ 6.65, LR− 0.26 [47]	0.10% (0.027–0.39%); 1 in 983	2.5% (1.7–3.7%)	38 (26–57)
1.01% (1 in 99); predicted at age 75–79 years, modelled [21]	MRI; published pooled LR+ 13.55, LR− 0.08 [50]	0.082% (0.020–0.33%); 1 in 1,226	12% (5.9–23%)	7 (3–16)
	MRI; derived LR+ 7.72, LR− 0.177, secondary scenario [45]	0.18% (0.11–0.31%); 1 in 554	7.3% (3.4–15%)	13 (6–28)
	Ultrasound; published pooled LR+ 6.65, LR− 0.26 [47]	0.26% (0.071–1.0%); 1 in 378	6.4% (4.3–9.2%)	15 (10–22)
2.0% (1 in 50); rate approached after age 60 years in five age-stratified surgical series, lower value [13]	MRI; published pooled LR+ 13.55, LR− 0.08 [50]	0.16% (0.041–0.65%); 1 in 614	22% (11–38%)	4 (2–8)
	MRI; derived LR+ 7.72, LR− 0.177, secondary scenario [45]	0.36% (0.22–0.62%); 1 in 277	14% (6.6–26%)	6 (3–14)
	Ultrasound; published pooled LR+ 6.65, LR− 0.26 [47]	0.53% (0.14–2.0%); 1 in 189	12% (8.3–17%)	7 (5–11)
3.0% (1 in 33); rate approached after age 60 years in five age-stratified surgical series, upper value [13]	MRI; published pooled LR+ 13.55, LR− 0.08 [50]	0.25% (0.062–0.98%); 1 in 405	30% (16–48%)	2 (1–5)
	MRI; derived LR+ 7.72, LR− 0.177, secondary scenario [45]	0.55% (0.33–0.93%); 1 in 183	19% (9.7–35%)	4 (2–9)
	Ultrasound; published pooled LR+ 6.65, LR− 0.26 [47]	0.80% (0.22–3.0%); 1 in 125	17% (12–23%)	5 (3–7)

LR+, positive likelihood ratio; LR−, negative likelihood ratio; MRI, magnetic resonance imaging. Post-test odds = pre-test odds × likelihood ratio; post-test probability = odds/(1 + odds). Published pooled likelihood ratios were used where reported [47,50]; both source meta-analyses pooled each measure separately with univariate random-effects models, so these ratios are not equal to those implied by the pooled sensitivity and specificity (for MRI, 22.5 and 0.10). For the synthesis that reported only sensitivity and specificity [45], likelihood ratios were derived as LR+ = sensitivity/(1 − specificity) and LR− = (1 − sensitivity)/specificity; these are secondary scenario calculations, not formal pooled estimates. Scenario envelopes are not formal 95% confidence or credible intervals: for published likelihood ratios they correspond to

the reported 95% confidence limits of each ratio, and for derived ratios to the extreme combinations of the marginal 95% limits of sensitivity and specificity. All values treat the baseline risk as known, although each baseline value is itself uncertain. Published likelihood ratios are applied with the target conditions defined by their source syntheses; the secondary scenario derived from [45] comes from pathologically heterogeneous malignant cohorts, so its application to all-sarcoma baseline risks is illustrative rather than strictly target-matched. Applying any all-sarcoma estimate to leiomyosarcoma alone is an approximation, because neither the baseline risks nor the likelihood ratios are specific to leiomyosarcoma; the values in this table should not be read as leiomyosarcoma-specific probabilities. The 0.39% baseline value is reported by its source as unexpected sarcoma, although all four events were leiomyosarcomas [17]. Most baseline values are partly post-assessment quantities and not true pre-imaging probabilities (Section 6); because prior assessment and a further imaging test are unlikely to be conditionally independent, the calculated residual risks may be underestimated. False-positive results per true positive = (1 − p)/p, where p is the post-test probability after a positive result.

Table S2. Study-level performance of the individual imaging, biomarker, score and biopsy studies.

Source	Design and population	Principal quantitative result	Principal limitation
Tamai et al., 2008 [37]	Target condition: Uterine sarcoma (five LMS, two ESS). Retrospective; 58 myometrial tumours from 43 patients, seven sarcomas	ADC 1.17 ± 0.15 × 10^{-3} mm^2/s for sarcoma, 1.70 ± 0.11 for degenerated leiomyoma and 1.62 ± 0.11 for normal myometrium; values overlapped with ordinary and cellular leiomyoma	Seven sarcomas; single centre
Kawamura et al., 2002 [65]	Target condition: Uterine sarcoma (seven cases). Prospective; transcervical needle biopsy in 435 patients; diagnostic-accuracy analysis of 372 biopsies from 351 patients (seven sarcoma-positive, 365 sarcoma-negative observations)	At the selected cut-off: sensitivity 100% (95% CI 59–100%), specificity 98.6% (95% CI 97.4–99.8%), PPV 58% (95% CI 30–86%), NPV 100.0% (95% CI 99.0–100.0%)	Seven sarcomas; biopsy-level rather than patient-level analysis, with repeat biopsies in some patients; mixed reference standard of surgical histology and at least two years of clinical follow-up; 100% point estimates are highly imprecise and do not establish perfect diagnostic performance
Goto et al., 2002 [55]	Target condition: LMS (ten cases). Prospective; 227 eligible patients, 10 LMS and 130 degenerated leiomyomas	LDH isozymes with dynamic contrast-enhanced MRI: sensitivity, specificity, PPV and NPV all 100%	A point estimate of 100% based on only ten malignant cases is highly imprecise and should not be interpreted as evidence of perfect diagnostic performance
Sato et al., 2014 [40]	Target condition: LMS. Retrospective, 1.5 T; 10 leiomyosarcoma lesions and 83 leiomyoma nodules, of which 41 intermediate-signal and 9 high-signal areas were measured	ADC 0.791 ± 0.145 versus 1.472 ± 0.285 (intermediate-signal areas, $p < 0.001$) and 1.100 ± 0.343 (high-signal areas, $p = 0.030$) × 10^{-3} mm^2/s; sensitivity 100%, specificity 94.0%, PPV 66.7%, NPV 100%	Ten lesions; PPV 66.7% even at the study's own prevalence
Nagai et al., 2014 and 2015 [58,59]	Target condition: Uterine sarcoma (15 cases). Retrospective; 63 patients, including 15 sarcomas	PRESS accuracy 84.1% with NPV 93.2%; revised score accuracy 93.7% with PPV 92.3% and NPV 94.0%	The revision was refitted on the derivation sample and is not an external validation
Przybyl et al., 2019 [61]	Target condition: Not applicable (benign leiomyoma only). Prospective; plasma from 12 women with leiomyoma	Tumour-derived copy-number alterations detected in six of 12 (50%)	Twelve patients; no sarcoma comparator group

Smadja et al., 2023 [66]	Target condition: Uterine sarcoma (11 cases). Prospective pilot; percutaneous biopsy with array comparative genomic hybridisation in 34 patients, 11 sarcomas	Accuracy 94% for microscopy alone and 100% combined; sensitivity, specificity and NPV 100%; no complications and no dissemination along the biopsy track	34 patients; single centre; point estimates of 100% based on only 11 sarcomas are highly imprecise and do not establish perfect diagnostic performance
Knipprath-Mészáros et al., 2023 [49]	Target condition: Masses with sarcomatous components, as defined by the source (16 cases). Prospective cohort; 545 patients	Basel sarcoma score above one point: sensitivity 93.8%, specificity 97.9%, PPV 57.7%, NPV 99.8%, AUC 0.95	Evaluated in the cohort in which it was developed; referral-level prevalence
Mahmood et al., 2023 [38]	Target condition: LMS versus degenerating leiomyoma and variants. Retrospective; histologically proven cases	Quantitative multiparametric MRI did not differ significantly between groups; concomitant necrosis and haemorrhage occurred only in leiomyosarcoma (two cases), and infiltrative margins in one leiomyosarcoma	Single-centre retrospective series; a non-significant quantitative result is not evidence of no difference
De Bruyn et al., 2024 [48]	Target condition: Uterine sarcoma (16 cases). Retrospective ultrasound cohort; 16 sarcomas and 91 leiomyomas	Median age 65 years (IQR 60–70) versus 48 (43–52); postmenopausal in 13 of 16 versus 15 of 91; irregular borders, non-uniform echogenicity and abundant vascularity common in sarcoma	Interobserver agreement for shape, border and echogenicity only fair
Kim et al., 2024 [41]	Target condition: Uterine sarcoma (44 cases). Four institutions; 44 sarcomas and 94 atypical leiomyomas	ADC 0.94 ± 0.30 versus $1.23 \pm 0.25 \times 10^{-3}$ mm²/s ($p < 0.001$); relative contrast ratio 8.16 ± 2.94 versus 4.19 ± 2.66 ($p < 0.001$); validation AUC 0.92 with quantitative DWI versus 0.78 without ($p < 0.001$)	Case-control assembly; odds ratios carry very wide confidence intervals
Yamanishi et al., 2025 [56]	Target condition: Uterine sarcoma as defined by the source (139 cases, including 80 LMS and 16 STUMPs). Fifteen centres; 139 sarcomas and 141 fibroids	Routine preoperative report sensitivity 77.7% with specificity 92.9%; two prespecified MRI-and-LDH algorithms 92.1%/72.3% and 82.0%/85.8%	Retrospective; case-control assembly
Vollmer et al., 2025 [60]	Target condition: LMS. Case-control validation; 90 leiomyosarcomas and 659 prospectively recruited leiomyomas	pLMS AUC 0.97 in the validation set; 0.960 versus 0.845 for a clinical-characteristics score in the comparison subset	Case-control assembly may overestimate apparent discrimination because of spectrum and design effects and limits transportability to unselected clinical populations
Stahlschmidt et al., 2026 [62]	Target condition: LMS. Prospective multicentre; 78 leiomyomas and 24 leiomyosarcomas	Cell-free RNA classifier AUC 0.868, sensitivity 0.732, specificity 0.813	Leiomyosarcoma-only malignant group; performance reported in cross-validation

ADC, apparent diffusion coefficient; AUC, area under the receiver operating characteristic curve; CI, confidence interval; DWI, diffusion-weighted imaging; IQR, interquartile range; LDH, lactate dehydrogenase; LMS, uterine leiomyosarcoma; MRI, magnetic resonance imaging; NPV, negative predictive value; PPV, positive predictive value; PRESS, preoperative sarcoma score; ESS, endometrial stromal sarcoma;

STUMP, smooth muscle tumour of uncertain malignant potential. Target conditions are as reported by the source. Reference numbers correspond to the reference list of the main manuscript. Prospective designs are named in the second column; all other entries are retrospective.

Table S3. Location within the manuscript of the elements addressed by the SANRA criteria for narrative reviews.

SANRA criterion	Where addressed
Justification of the article's importance for the readership	Section 1, paragraphs 1 to 5
Statement of concrete aims or formulation of questions	Section 1, final paragraph
Description of the literature search	Section 2: databases, search terms, language restriction, prioritised evidence types, supplementary targeted searches of interventional radiology and device guidance, and handling of overlapping and discordant evidence
Referencing	Throughout; citations placed immediately after the statement they support, numbered in order of first appearance
Scientific reasoning	Section 3.1 (reconciliation of discordant prevalence estimates), Section 4.4 (tissue properties underlying imaging signals), Section 5.2 (two disagreeing meta-analyses), Section 6 (published versus derived likelihood ratios; exploratory reconstruction from a consecutive cohort under stated assumptions), Section 7.1 (three discordant morcellation datasets), Section 9.1
Appropriate presentation of data	Tables 1 to 3, Tables S1 and S2, Figures 1 to 3, and Sections 3, 5 and 6, where effect estimates are given with confidence intervals and absolute risks with their denominators

This table maps the manuscript against the SANRA framework for transparency. It records where each element is addressed and assigns no score; SANRA is a quality framework for narrative reviews and is not a systematic-review methodology.
